# Shifting faunal baselines through the Quaternary revealed by cave fossils of eastern Australia

**DOI:** 10.7717/peerj.6099

**Published:** 2019-01-22

**Authors:** Gilbert J. Price, Julien Louys, Garry K. Smith, Jonathan Cramb

**Affiliations:** 1 School of Earth and Environmental Sciences, The University of Queensland, Brisbane, QLD, Australia; 2 Australian Research Centre for Human Evolution, Griffith University, Brisbane, QLD, Australia; 3 Newcastle and Hunter Valley Speleological Society, Newcastle, NSW, Australia

**Keywords:** Marsupials, Megafauna, Australia, Conservation, Caves, Palaeontology, Extinction, Extirpation, Mammals, Fossils

## Abstract

Fossils from caves in the Manning Karst Region, New South Wales, Australia have long been known, but until now have never been assessed for their palaeontological significance. Here, we report on late Quaternary faunal records from eight caves in the region. Extinct Pleistocene megafaunal taxa are recognised in two systems and include giant echidnas (Tachyglossidae gen. et sp. indet.), devils (*Sarcophilus laniarius*), koalas (*Phascolarctos stirtoni*), marsupial ‘lions’ (*Thylacoleo carnifex*), and kangaroos (*Macropus giganteus titan*). Some caves contain skeletal remains of introduced exotics such as sheep and dogs, but also provide a rich record of small-bodied native species including Eastern Bettongs (*Bettongia gaimardi*), Eastern Chestnut Mice (*Pseudomys gracilicaudatus*), and White-footed Rabbit Rats (*Conilurus albipes*). These endemics are either locally extirpated or have suffered total extinction in the historic period. Their skeletal and dental remains were recorded as unmineralised surface specimens in the caves, indicating that they are recent in age. Extant populations have never been recorded locally, thus, their probable loss from the region in historic times had gone unnoticed in the absence of palaeo-evidence. Our findings suggest that the supposed habitat tolerances of such species have been substantially underestimated. It is highly likely that modern populations have suffered niche contraction since the time of European colonisation of the continent. The local extirpations of several species of digging mammal has likely led to decreased functionality of the current ecosystem.

## Introduction

Caves are a critical source of vertebrate fossils (Bacon et al., 2004; Lundelius, 2006; Jass & George, 2010; McFarlane, 2013; Louys et al., 2017; Price et al., 2017; Hawkins et al., 2018; Louys, 2018). The preservation of vertebrate assemblages within a cave are governed by a complex set of factors including local geology, hydrology, biogeography, and cave topology and topography (Simms, 1994; Jass & George, 2010; Louys et al., 2017). Heterogeneous distribution of vertebrate assemblages from different caves and cave types can influence interpretations of past biogeographic distributions (Jass & George, 2010).

Understanding past biogeographical patterns is essential for developing accurate projections of species distributions across the landscape now and into the future, information fundamental for conservation efforts under a rapidly changing climate (McGuire & Davis, 2014). As such, sampling of vertebrate records from different types of caves across a variety of regions is a crucial first step for conservation palaeobiology approaches (Fusco, McDowell & Prideaux, 2016).

In Australia, the first fossils reported in the scientific literature came from the Wellington Caves of New South Wales (Owen, 1838). Since that time, palaeontological investigations of caves have been pivotal in providing insights into the origins of Australia’s biota, especially across the mid- to late Cenozoic. The oldest Australian cave deposits are from the Riversleigh World Heritage Area of northwest Queensland (Archer et al., 2006), the majority of which date to the Miocene. In contrast, the younger end of the fossil record, the Quaternary (last 2.6 Ma), is best represented by caves in the limestone karsts that fringe the modern coastline of the continent.

Significantly, however, there are numerous gaps in the spatial distribution of caves dating to this period. By far the most intensively sampled caves are those in the southern margins of the continent (e.g. southwest Western Australia, Nullarbor, and Naracoorte), with the eastern coast being less explored, particularly in recent times. This is not necessarily through lack of fossils in caves in the east, but rather the challenges in conducting palaeontological survey work in the heavily karstified, hard-rock and geologically older limestones in the region, vs the younger, soft-rock limestones (Grimes, 2006) in the south and west.

**Figure 1:**
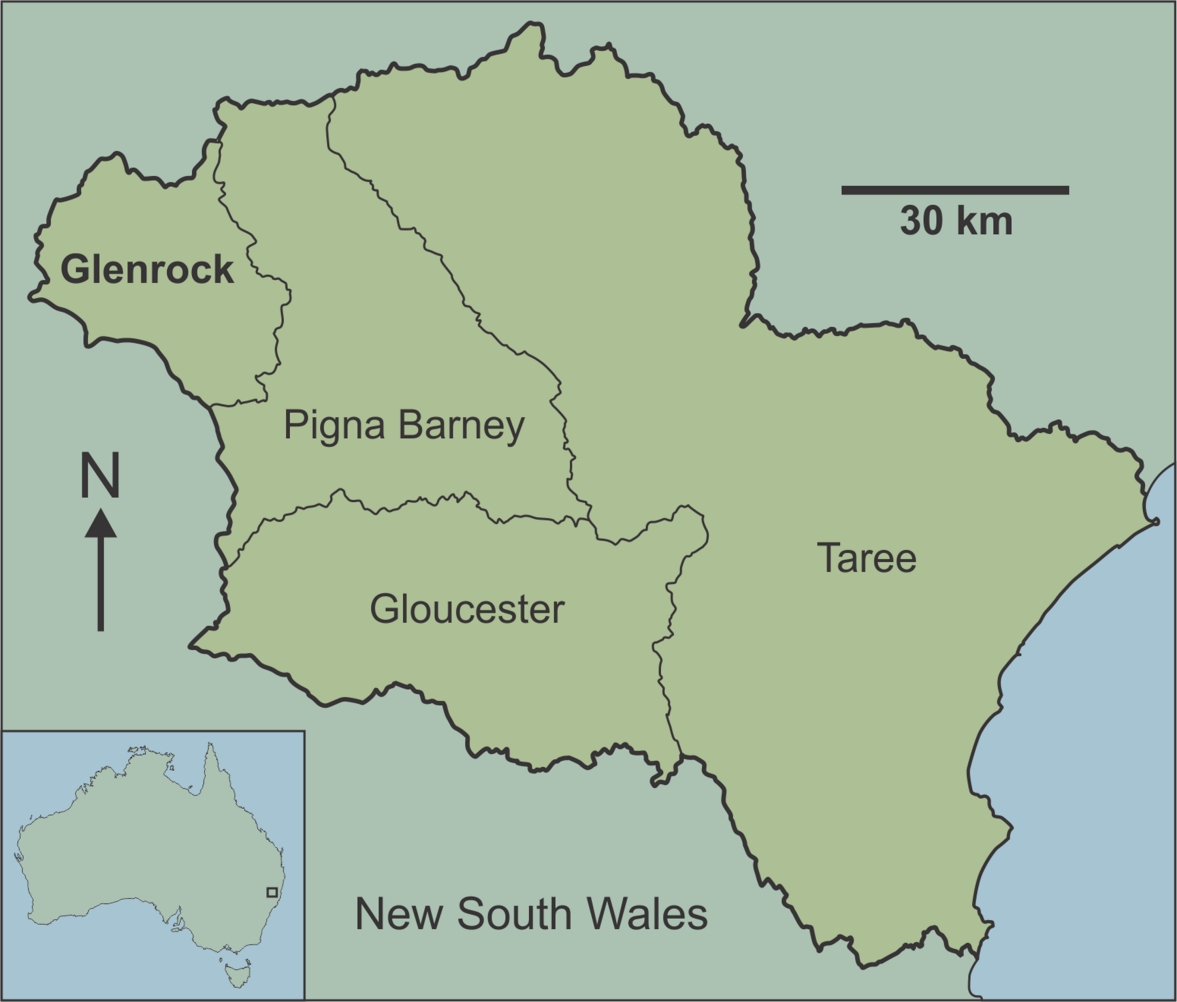
Map of Australia showing location of the Manning Karst Region and contained karst areas including Glenrock (following Smith, 2011a).

Here, we provide the first detailed report of fossil-bearing caves in the Manning Karst Region of eastern New South Wales (Fig. 1). Although fossil bones have been reported in some of the caves previously (Pinnock, 1987), they have not been the subject of detailed palaeontological investigation. The aim of this study is to estimate vertebrate diversity preserved in the Glenrock cave systems of the Manning Karst Region. Our study highlights new records of fauna for the region, with implications for understanding past distributions of extinct and extirpated species in the temperate zone of eastern Australia, a region that has been densely populated and significantly impacted since European arrival.

## Geographic and Geologic Settings

The Manning Karst Region comprises four distinct karstic zones (Fig. 1) in a remote region of eastern New South Wales (Smith, 2011a). Caves in the region are mostly located on private property and have rarely been visited, meaning that they are in relatively pristine condition. Speleologists have known of the existence of caves in the area since the 1980s. Between 1983 and 1987 a concerted effort by cavers identified approximately 108 caves. Exploration slowed at this point, then from 1994 to the present day the Newcastle and Hunter Valley Speleological Society (NHVSS) has continued the push to discover additional caves in the area (Smith, 2011b, 2012, 2015). Today there are over 157 known caves and several with depths approaching or greater than 47 m and survey lengths up to a couple of hundred metres.

The limestone caves discussed here are from Glenrock (Fig. 1), a private cattle station accessible only via permission from the land manager. Caves at the station occur at around 650 m above sea level. The dominant vegetation is eucalypt forest with grassy understorey, although there has been substantial habitat clearing in the region in historic times (the term ‘historic’ is used informally throughout this paper to denote the time since European colonisation of the continent). The karst area is notorious for ‘foul air’ in the deeper caves (‘foul air’ is an atmosphere that contains greater than 0.5% CO_2_ and/or lower than 18% O_2_ by volume) (Smith, 1996, 2011a, 2012) which can be fatal.

The caves occur within limestone of the Silver Gully Formation, part of the Tamworth Group. Deposition of the limestones occurred in shallow seas in reef complexes that were part of the Tamworth Trough (Lishmund, Dawood & Langley, 1986). Biostratigraphic considerations of radiolarians and conodonts place the age of the limestone as upper Emsian, ca. 400 Ma (Aitchison et al., 1999). At Glenrock, the limestone crops out over a distance of approximately 10 × 2 km along the fault-orientated Orham Creek. The limestones have been tilted close to 90°. The caves are joint controlled; thus most are vertical systems. Glenrock caves are generally devoid of speleothems (Smith, 2011a).

## Methods

Many of the caves in the area are readily discernible on the landscape, consisting of vertical openings at ground level, up to four m in diameter, commonly with fig trees growing adjacent to them. However, since 1994 many of the newly discovered caves have been manually exposed by digging out cave entrances. Depressions (or ‘dolines’) in the landscape, commonly with vegetation growing over the surface, often act as sumps that drain meteoric water into caves below. These depressions are probed with ridged steel rods and iron digging bars; if they easily penetrate into the ground, a cave is indicated and the entrance can then be excavated.

Some of the Glenrock caves can be entered via free-climbing, but others require the use of ropes to access them. On some small pitches, it is practical to use wire rope caving ladders and safety belays.

During initial surveys of new caves, those that contain visible bones were labelled as such on survey maps and notes added in the cave description. Fossil-bearing caves reported in this study were originally discovered by the NHVSS and Hills Speleology Club.

Cave area codes and numbers follow Pinnock (1987) where ‘GR*n*’ refers to ‘Glenrock Cave *n*’ (e.g. cave GR10). Each cave from which bones were collected are cross-referenced with Australian Age of Dinosaur fossil locality numbering, where additional details, including map coordinates, are available. GPS coordinates are not given in this paper as the caves are on private property (to protect them from potential vandals and trespassers on private land), but are available to *bona fide* researchers upon request to the Australian Age of Dinosaurs Museum.

Fossil surveys within the caves were generally conducted by exploring various caverns and subterranean deposits, visual inspection, and documentation of surface materials rather than detailed excavations. Representative samples of most species (especially Australian endemic taxa) were collected and are now curated in the collections of the Australian Age of Dinosaurs Museum, Winton, Queensland.

Where appropriate, fossils were measured with digital callipers with precision reported to 0.1 mm. For agamid lizards, dental nomenclature follows Hocknull (2002). For marsupials, dental nomenclature follows Luckett (1993) where the unreduced tooth formula is P1–3 and M1–4 in both lower and upper dentitions (upper teeth are denoted here using superscript numbering, while lower teeth are indicated by subscript numbering; e.g. M^1^ vs M_1_ for upper and lower first molars, respectively). Dental cusp morphology for marsupials follows Archer (1978), except for the hypocone which is referred to as the metaconule following Tedford & Woodburne (1987, 1998). Dental cusp morphology for rodents follows Cramb, Price & Hocknull (2018), as modified from Musser (1981). Because most species are relatively well-known, descriptions of fossil fauna have been limited to diagnostic characters in most cases. Voucher specimens were recovered and registered in the museum collection for most native species, but non-natives (e.g. dogs, sheep) were simply observed in the field and their occurrence within the caves noted.

## Results and Interpretation

### Speleology

#### GR1 Fig Tree Cave (AODL0253)

Fig Tree Cave (Fig. S1) has an 11 m deep entrance shaft that measures 5 × 2 m at the surface. A ledge of jammed rocks is encountered five m down, then a further six m of vertical shaft occurs before reaching a relatively flat earth and broken rock floor. From this point, phreatic passages can be followed short distances to enter the two avens (vertical shafts), each about 13 m high; both contain red flowstones and stalactites that are mostly inactive. Bone deposits are located in the soft silt under travertine at the deepest part of the cave, as well as on the wall of one passage. The total vertical depth of the cave is 18.5 m and survey passage length is 51 m.

#### GR2 Clunk, Clunk, Bang, Pot Cave (AODL0254)

This cave (Fig. S2) is located in a grove of fig trees 20 m from GR1. The entrance measures 2 × 1 m at the top of an 11.5 m shaft. The restricted entrance suggests that the cave has less potential to be a natural trap for large animals. Several large fig tree roots hang down the full length of the cave’s shaft and continue into the silt and broken rock floor at the bottom of the cave. The single chamber at the bottom measures just five m diameter, with little chance of extension without extensive excavation. Faunal remains were found in small niches away from the main entrance.

#### GR5 Ewe Beaut Cave (AODL0255)

This cave is located on grassed, gently sloping terrain (Fig. S3). There are two trees growing at the side of the 11 m vertical, solution tube entrance. The entrance measures 4 × 2 m at the surface. The cave has a high potential to act as a natural animal trap, as is obvious by the large number of bones that litter the earth and broken rock floor. A single chamber at the bottom of the shaft measures 7 × 5 m, with little chance of extension. On the western side of the chamber, a four m high section of red flowstone decorates the wall.

#### GR17 Red-naped Cave (AODL0256)

GR17 (Fig. S4) is located in lightly timbered and grass terrain; however, the immediate surrounds of the entrance is devoid of vegetation. The smooth bedrock around the entrance slopes abruptly into the vertical 20 m deep solution shaft. The shaft at the entrance measures 3 × 4 m and opens out to a split level single chamber, approximately nine m diameter at the bottom. The rock-pile floor slopes from west to east and ends in a dirt and rubble floor. The lowest point of Red-naped Cave contains foul air.

The most fossil-rich deposit is located in the northeast side of the main chamber. The clay deposit that contains the bones is trapped in a solution hollow dissolved from the solid bedrock approximately one m above the present sediment floor of the cave. The maximum depth of the cave is 24 m and survey length is 43 m.

#### GR18 Westgate Cave (AODL0257)

The two entrances to this cave are approximately one m apart under the canopy of a fig tree (Fig. S5). Both entrances are one m in diameter and either can be used to descend the 10 m pitch into a chamber below (which measures 8 × 5 m) that contains abundant speleothem formation. A six m tall tree root runs from the ceiling to the rock-fill floor. Two additional pitches of four and three m, respectively, lead to lower levels of the cave. Foul air is usually encountered beyond the first pitch. The dirt floor in the lower west section contains sheep bones and bat guano.

#### GR36 Lonesome Lair (AODL0258)

This small cave has little chance of being a natural animal trap (Fig. S6). Rather, it is more likely used as a residence or safe haven for animals. The entrance is located amongst fractured bedrock just above the level of the modern creek. The small gently sloping entrance passage leads to a chamber 3.5 m high by two m diameter. Several short passages of one and two m in length lead off the main chamber. Bones from wallabies and wombats were previously identified in the cave. No foul air was recorded in this cave.

#### GR92 Leaf Beetle (AODL0259)

GR92 has a small entrance above a vertical drop of three m to a debris floor littered with bones (Fig. S7). There is a small amount of flowstone. No foul air was recorded in this cave.

#### GR124 Death Trap Cave (AODL0260)

The entrance to this cave (Fig. S8) was completely closed prior to its discovery in 2010. The entrance is permanently closed with iron sheeting when not being explored. The very small entrance measures 0.8 × 0.4 m and descends vertically two m into a tight spiralling passage with several drops of about two m. A short crawl then leads into a chamber, 10 × 6 × 2 m high. A 6.5 m aven is located on the southwestern side of the chamber. To the east, a 0.6 m crawl leads to a low chamber that contains a two m tall leaning column near a five m deep, very narrow (impenetrable) rift with air flowing from below.

In the centre of the first chamber there are two holes leading to chambers below. The small chamber below this free-climb contains numerous large bones as well as an excellent example of a three m long rootsicle (tree root covered in thick calcite). From this small chamber, a low crawl passage leads to a four m climb down the side of a chamber that measures 6 × 4 × 2.7 m high, and contains an active flowstone wall.

The roof height drops to 1.2 m and a trench between adjacent fine clay banks may be followed for six m in an easterly direction. The cave height above the clay banks is just 0.2–0.3 m for a length of three m either side of the erosion trench. Several bones are visible protruding from the sediment in that part of the cave. The trench leads into a climbable passage that descends a further four m to a narrow earth floor chamber which sometimes contains foul air. This chamber also contains speleothems.

Death Trap Cave is best described as having two main levels, although there are some additional pitches on each level. The majority of the fossil bones are found in the lower level. The whole cave may be explored without ropes or ladders, however, the entrance and initial passage is tight. Death Trap Cave has a total depth of 33 m and a survey length of 90 m.

### Fauna

#### Anura

Anura gen. et sp. Indeterminate

**Figure 2:**
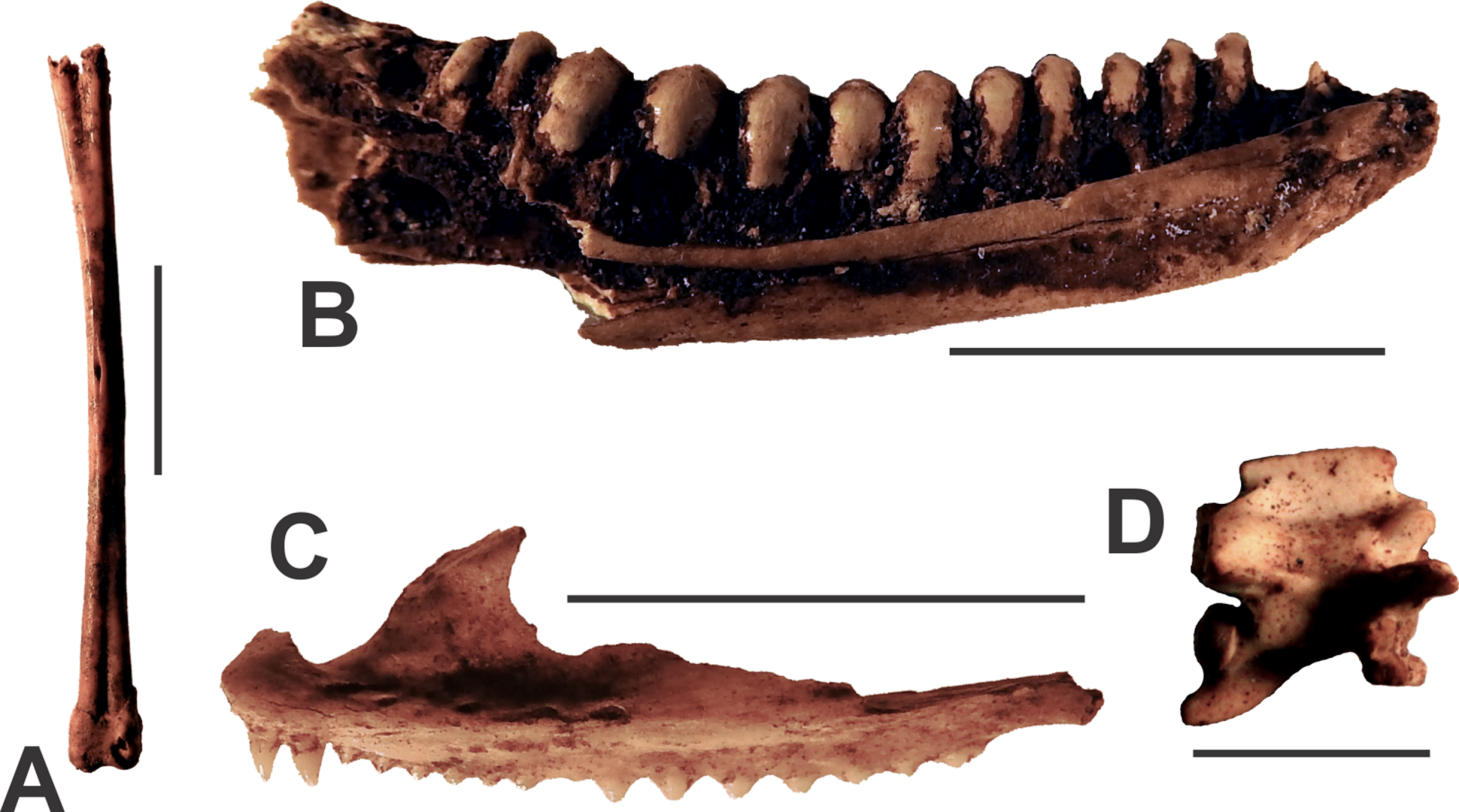
Herpetological taxa from the Manning Karst Region. (A) Anuran tibiofibula (AODF0907). (B) *Tiliqua scincoides* left dentary (AODF0908). (C) *Lophognathus gilberti* left maxilla (AODF0909). (D) Elapid vertebra (AODF0910). Scale bars = 10 mm.

Frog bones were recorded as surface specimens in some caves (Table 1) including various limb bones such as tibiofibulae (AODF0907; Fig. 2A). The assemblages lack ilia, the most useful skeletal element commonly used for the taxonomic identification of frogs (Price, Tyler & Cooke, 2005). The bones are generally white in colour, unmineralised, and are most likely modern.

**Table 1:** Species lists for caves of the Manning Karst Area.

Taxon	GR1 Fig Tree Cave	GR2 Clunk, Clunk, Bang, Pot Cave	GR5 Ewe Beaut Cave	GR17 Red-naped Cave	GR18 Westgate Cave	GR36 Lonesome Lair	GR92 Leaf Beetle	GR124 Death Trap Cave
Anura gen. et sp. indet.				X				X
*Tiliqua scincoides*								X
*Lophognathus gilberti*			X	X	X			
Elapidae gen. et sp. indet.		X	X	X				
*Tachyglossus aculeatus*	X							
Tachyglossidae sp. gen. et sp. indet.				X*				
*Antechinus flavipes*		X						
*Sarcophilus harissii*	X							
*Sarcophilus laniarius*				X*				X*
*Isoodon obesulus*	X	X						X
*Phascolarctos stirtoni*				X*				
Vombatid gen. et sp. indet.	X			X*				X*
*Thylacoleo carnifex*				X*				
*Trichosurus* sp. indet.	X							X*
*Bettongia gaimardi*		X						X
*Notamacropus rufogresius*				X				
*Macropus giganteus giganteus*	X		X				X	
*Macropus giganteus titan*				X*				
*Petrogale penicillata*				X*		X		X
*Wallabia bicolor*				X				
Microchiropteran gen. et sp. indet.	X				X			
*Conilurus albipes*	X							
*Pseudomys gracilicaudatus*					X			
*Pseudomys oralis*	X				X			
*Rattus* sp. (*R. tunneyi* or *R. fuscipes*)	X	X		X	X			
Muridae *ge. et. sp. indet.*					X			
*Vulpes vulpes*				X				
*Canis familiarus*	X		X					
*Ovis aries*			X	X				
*Oryctolagus cuniculus*						X		

**Notes:**

X, presence.

* Mineralised specimen.

#### Scincidae

Tiliqua scincoides

A Blue-tongue Skink is represented by a single left mandible (AODF0908; Fig. 2B) found in Death Trap Cave. It was identified on the basis of its large size, possession of a closed Meckel’s groove, and presence of large, uniform, hemispherical-conical teeth. The specimen was collected from the surface of the lower section of Death Trap Cave. It is white in colour and unmineralised, suggesting that it is modern. Blue-tongue Skinks are common in eastern Australia today.

#### Agamidae

Lophognathus gilberti

Maxillae (e.g., AODF0909; Fig. 2C) and associated dentaries are referred to Gilbert’s Dragon and were recorded in three caves (Table 1). The dorsal maxillary process is constricted superiorly and broad superiorly. The suborbital margin is relatively large and the narial process is tall and robust. It possesses two pleurodont teeth of equal size and 14 acrodont teeth (seven small, seven large). They were surface specimens, slightly discoloured, and unmineralised, suggesting that they are modern. *Lophognathus gilberti* occurs in eastern Australia today.

#### Elapidae

Elapidae gen. et sp. indet.

Several isolated vertebrae (e.g. AODF0910; Fig. 2D) and associated rib bones, as well as a near-complete desiccated skeleton of elapid (venomous) snakes were recovered as surface specimens in some of the caves (Table 1). Their identification as elapids was based on the vertebrae which possess long, acute accessory processes, and a hypapophysis that arise near the cotyle tip and extend posteriorly for approximately half the length of the condyle. The specimens are similar in size of Brown (*Pseudechis australis*) and Red-bellied Black snakes (*Pseudechis porphyriacus*), but could not be identified below the family level. The fully skeletonised specimens were found on the surface of the cave floor. The association of fragile ribs suggests that they were minimally disturbed prior to collection. They are generally white in colour and unmineralised, indicating that they are modern.

#### Tachyglossidae

Tachyglossus aculeatus

**Figure 3:**
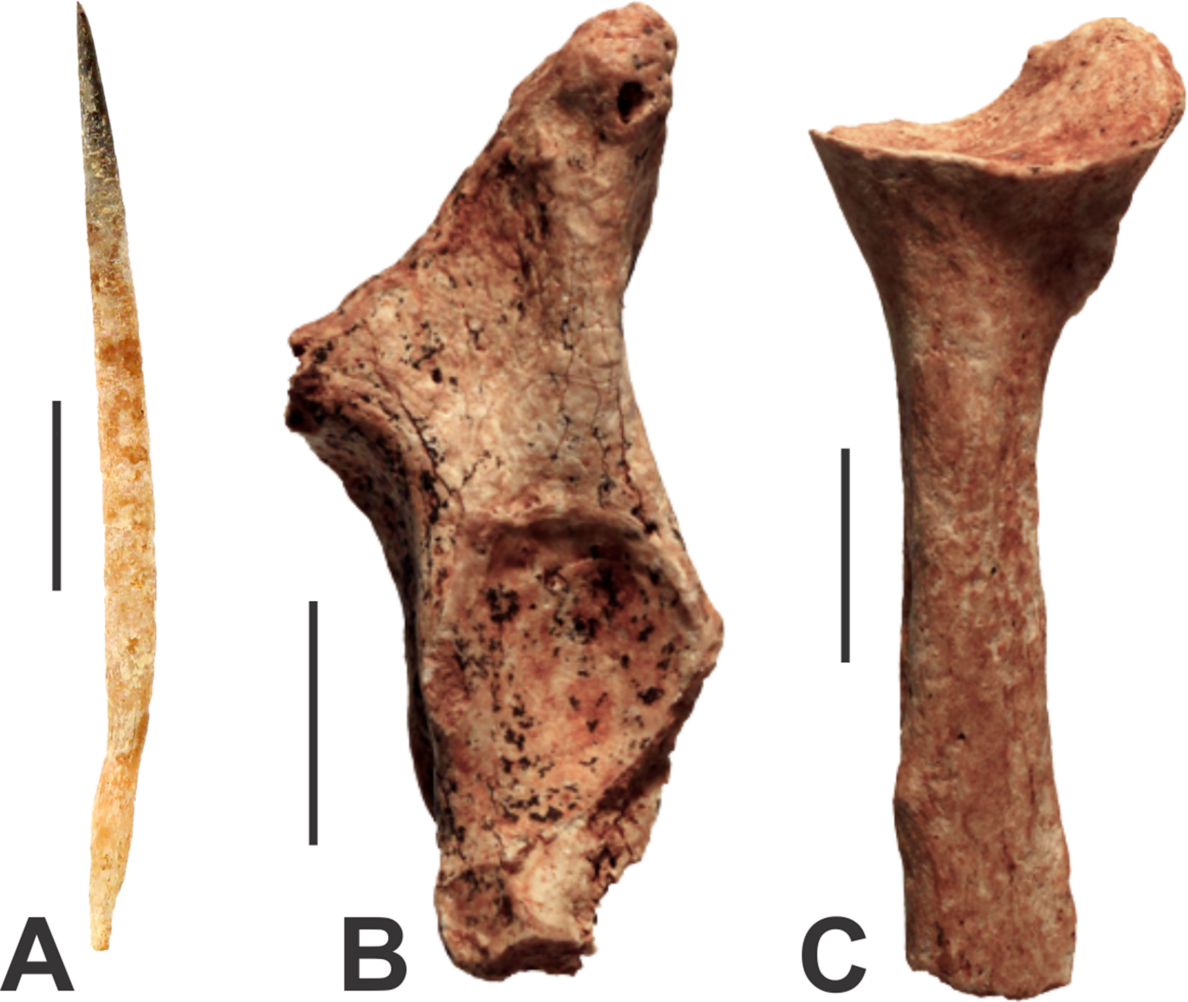
Monotremes from the Manning Karst Region. (A) *Tachyglossus aculeatus* quill (AODF0911). (B) Tachyglossidae gen. et sp. indet. left proximal ulna (AODF0912). (C) Tachyglossidae gen. et sp. indet. right proximal radius (AODF0913). Scale bars = 10 mm.

There are two very different-sized species of echidna present in the caves at Glenrock. The modern Short-beaked Echidna (*Tachyglossus aculeatus*) is represented by a quill measuring 50 mm in length (AODF0911; Fig. 3A) from Fig Tree Cave. Echidna quills are composed of keratin, a biological material susceptible to rapid decay. The specimen is well-preserved and unmineralised, suggesting that it is modern.

Tachyglossidae gen. et sp. indet.

A right ulna (AODF0912; Fig. 3B) and left radius (AODF0913; Fig. 3C) are referrable to a species of giant echidna. The radius (39.9 mm long) is broken close to the proximal end, just below the trochlear notch. The olecranon process has dual processes, a higher one that spreads posteromedially and a lower one that is angled anterolaterally. The anterolaterally directed process is well-developed, but is broken and missing the tip. The trochlear notch is a simple oval-shaped depression 12.3 mm across and differs from *Tachyglossus* by being more circular rather than ovate. The radius is also represented by the proximal end (46.1 mm long); it is clear that the bone would have been long and straight, and would have been appressed against the ulna. The head of the radius is broad and slightly cup-shaped for articulation with a bulbus condyle of the distal humerus. Both fossil specimens are substantially larger than the equivalent limbs of *Tachyglossus*. Two genera of giant echidna have been recognised in the Pleistocene deposits of Australia: *Megalibgwilia* and ‘*Zaglossus*’, the latter of which is likely distinct to modern *Zaglossus* (long-beaked echidnas of New Guinea) and also the largest-known echidna (Griffiths, Wells & Barrie, 1991). AODF0912 and AODF0913 are too fragmentary to be confidently referred to either taxon, but they are certainly not *Tachyglossus*. The specimens are mineralised suggesting considerable antiquity. Both specimens were found in Red-naped Cave within a deposit that contains other extinct megafaunal taxa.

#### Dasyuridae

Antechinus flavipes

**Figure 4:**
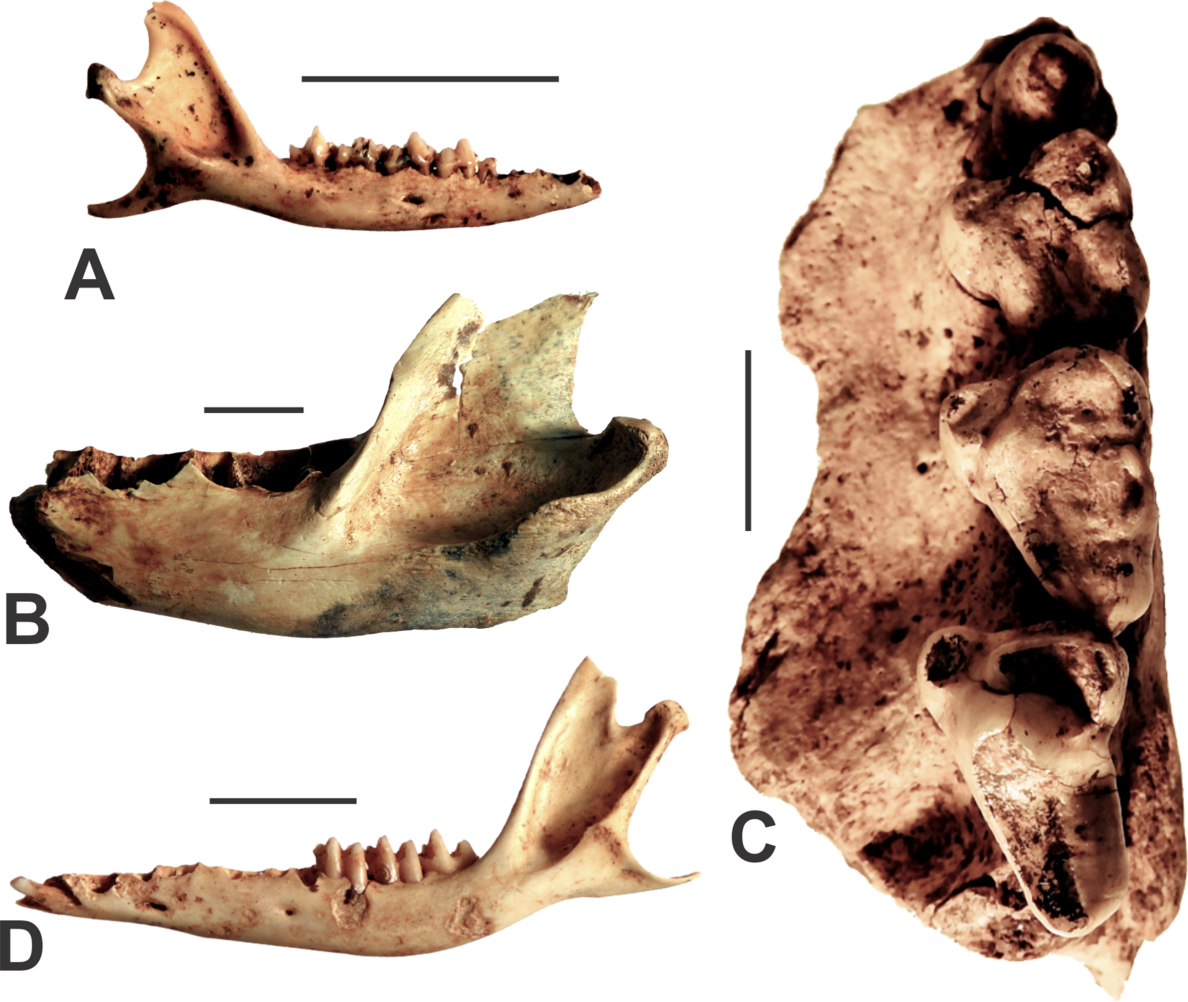
Agreodonts from the Manning Karst Region. (A) *Antechinus flavipes* right mandible (AODF0914). (B) *Sarcophilus harrisii* left dentary (AODF0915). (C) *Sarcophilus laniarius* left maxilla (AODF0917). (D) *Isoodon obesulus* left mandible (AODF0918). Scale bars = 10 mm.

A right mandible (AODF0914; Fig. 4A) from GR2 is referred to the Yellow-footed Antechinus. It is identified based on its small size (relative to other dasyurids), possessing a crowded premolar row, and a heavily reduced P_3_ that retains two roots. The specimen is whitish in colour and was found on the surface suggesting that it is modern. Yellow-footed Antechinuses are common in eastern Australia.

Sarcophilus harrisii

Fossils of marsupial devils were found in several of the caves (Table 1), although there are two clear size morphs present. The smaller-bodied species, *Sarcophilus harrisii* (Tasmanian Devil), is represented by a left mandible that is missing its teeth (AODF0915; Fig. 4B). Its identification is based on its large size (relative to other dasyurids), being robust (as opposed to slender as in *Thylacinus*), and possessing a low condyle in approximately the same plane as the tooth row. Although found on the surface of a deposit in Fig Tree Cave, the specimen is mineralised and a calcite crust had formed on the bone (although now removed following preparation), suggesting some antiquity. *S. harrisii* suffered extinction on the Australian mainland around 3,000–4,000 years ago (White et al., 2018). Thus, that timeframe indicates a minimum age for this specimen.

Sarcophilus laniarius

A very large fossil devil is represented by several specimens including an isolated lower canine (AODF0916), a partial left maxilla with P^3^–M^1–3^ (AODF0917; Fig. 4C), as well as broken and isolated molar teeth. The dentition is similar in gross morphology to modern *Sarcophilus*, but is extremely large in comparison (Table 2). The canine was found on the surface in Death Trap Cave, while the maxilla was recovered from a shallow, clay-rich deposit (that also included specimens of other megafaunal taxa) in Red-naped Cave. The youngest reliable date of *S. laniarius* on the continent is ca. 50 ka (Price et al., 2009b) while the oldest is ca. 500 ka (Prideaux et al., 2007). Thus, the Glenrock specimens are likely middle-late Pleistocene in age.

**Table 2:** Dimensions of *Sarcophilus laniarius* teeth from the Manning Karst Area (AODF0917) and comparative data from extant populations of *S. harissii* from Tasmania (mean, range; data from Dawson (1982)).

Tooth	Length	Width
P^3^	7.5 (6.4, 6.0–6.8)	6.8 (5.8, 5.3–6.3)
M^1^	11.5* (11.2, 10.7–12.0)	10.3 (9.1, 8.6–9.7)
M^2^	15.4 (12.1, 11.5–13.0)	11.0 (9.9, 8.3–10.2)
M^3^	15.1 (11.9, 11.0–12.4)	11.1 (9.8, 9.3–10.3)

**Notes:**

All measurements in mm.

* Minimum estimate due to interstitial wear from posterior tooth.

#### Peramelidae

Isoodon obesulus

The Southern Brown Bandicoot is represented by several specimens including mandibles (e.g., AODF0918; Fig. 4D) from adult individuals, most of which are missing teeth. They are identified as *Isoodon obesulus* on the basis of possessing a steeply inclined vertical ramus (ca. 115°) relative to the horizontal ramus. This feature clearly distinguishes them from other peramelids such as *Perameles* (long-nosed bandicoots). The specimens are intermediate in size between the large *I. macrourus* and smaller *I. auratus*. Three of the eight caves surveyed contained skeletal elements of the Southern Brown Bandicoot (Table 1). All specimens were found on the surface of cave floors or within exposed crevices. The bones are white and unmineralised, suggesting that they are modern.

#### Phascolarctidae

Phascolarctos stirtoni

**Figure 5:**
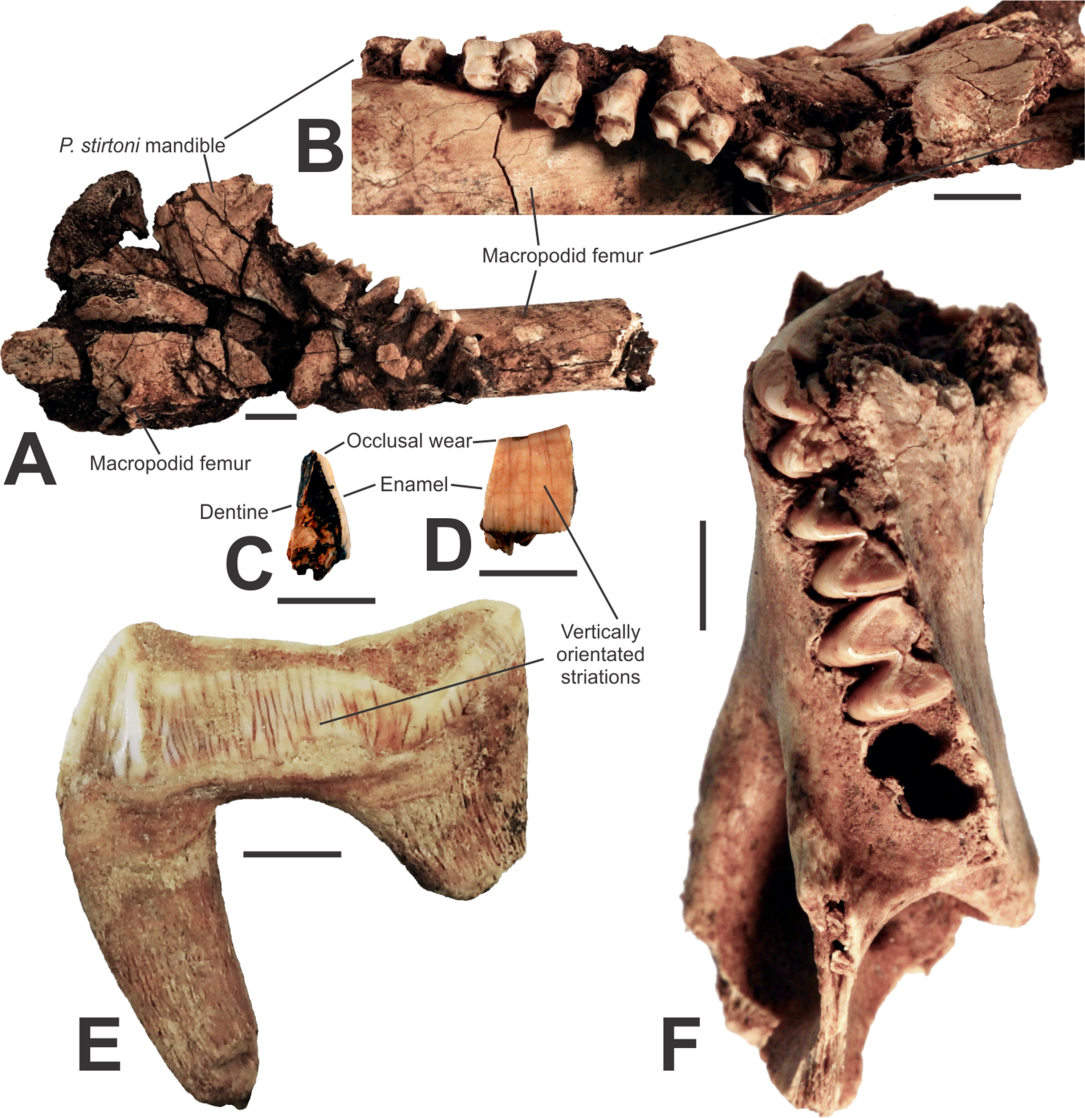
Vombatomorphians from the Manning Karst Region and a comparative specimen. (A and B) *Phascolarctos stirtoni* right mandible (AODF0920) in lateral and occlusal view, respectively. (C and D) *Thylacoleo carnifex* premolar fragment (AODF0922) in cross-section and enamel view, respectively. (E) *Thylacoleo carnifex* upper premolar (SAMP24101) from Goulden’s Hole, South Australia. (F) *Vombatus* sp. indet. left mandible (AODF0921). Scale bars = 10 mm.

A fossil lower right mandible (AODF0920; Figs. 5A and 5B) with P_3_–M_1–4_ is referred to the megafaunal Stirton’s Koala, *Phascolarctos stirtoni*. In gross morphology, the specimen is similar to the extant Koala, *Phascolarctos cinereus*. However, teeth of AODF0920 are up to 30% larger than the average size of equivalent dentition in the extant species and several are outside the entire size range of modern specimens (Table 3). The teeth are also smaller than the extremely large Pleistocene *Phascolarctos yorkensis* (see Table 3 of Pledge, 1992), the largest-known koala. The specimen is heavily mineralised and was substantially encrusted in calcite prior to preparation (it remains cemented to a macropodid femur and could not be removed without damaging the specimen. It is important to note that the calcite is impure and not suitable for radiometric dating, such as the U–Th method, e.g., Price et al., 2013). The specimen is from Red-naped Cave, in the same clay-rich deposit as the giant devil, AODF0917. The youngest geological age of *Phascolarctos stirtoni* is ca. 50 ka (Price et al., 2009b) whilst the oldest records are ca. 500 ka (Price, 2008). This suggests that the deposit is mid-late Pleistocene, and thus in agreement with the age suggested by the presence of the giant devil.

**Table 3:** Dimensions of *Phascolarctos stirtoni* teeth from the Manning Karst Area (AODF0920) and comparative data from extant populations of male *Phascolarctos cinereus* from Victoria, Australia (mean, range; data from Black, Louys & Price (2014) and Brewer et al. 2018)).

Tooth	Length	Anterior width	Posterior width
P_3_	NA	Missing	4.9 (4.0, 3.3–4.2)
M_1_	8.7 (7.6, 7.1–8.2)	5.8 (5.2, 4.7–5.4)	6.5 (5.4, 5.2–5.9)
M_2_	9.7* (8.1, 7.7–8.6)	6.2 (5.5, 4.8–5.9)	6.1 (5.3, 5.0–5.8)
M_3_	9.4 (7.9, 7.6–8.4)	6.1 (5.4, 5.0–5.8)	5.8 (5.1, 4.6–5.6)
M_4_	9.7 (8.1, 7.3–8.5)	6 (5.3, 4.9–6.1)	5.4 (4.7, 4.4–5.2)

**Notes:**

All measurements in mm.

* Approximate.

#### Thylacoleonidae

Thylacoleo carnifex

A small, fossilised tooth fragment (AODF0922; Figs. 5C–5D) is wedged-shaped and includes some vertically orientated, striated enamel on one side and exposed dentine on the other. The specimen is from a relatively large-bodied organism, but is not referable to the Diprotodontoidea (giant wombat-like marsupials) as the enamel is too thin (ca. 0.8 mm) relative to the size of the fragment. Macropodoidea and Vombatidae are excluded as the tooth fragment is too broad and flat to be part of either a kangaroo’s or wombat’s dentition, respectively. Only marsupial ‘lions’ possess such striations in dental enamel, especially on the upper and lower thirds premolars (e.g., SAMP24101, Fig. 5E). A mastication-related, obliquely angled wear surface on the tooth fragment is typical of the lingual side of upper and lower P3s in *Thylacoleo carnifex*. Given that the wear surface is indicative of the occlusal face of the tooth, the height of the crown is similar to that in *Thylacoleo carnifex*. Thus, we consider the specimen is a fragment of a third premolar of *Thylacoleo carnifex. Thylacoleo carnifex* is the largest-bodied species within Thylacoleonidae, weighing up to 160 kg (Wroe et al., 1999). Of note, the specimen is from the same deposit in Red-naped Cave that produced other megafaunal taxa. The youngest fossil record of *Thylacoleo carnifex* is ca. 48 ka (Prideaux et al., 2010), while the oldest is ca. 500 ka (Prideaux et al., 2007), thus, is in agreement with a probable age of mid-late Pleistocene for the deposit.

#### Vombatidae

*Vombatus* sp. indet.

Numerous fragmented and isolated teeth, as well as a partial lower left mandible (AODF0921; Fig. 5F) were found in three of the region’s caves. The species is a medium-sized wombat, within the size range of the extant *Vombatus ursinus*. Species of *Vombatus* differ from those of the other extant genus of wombat, *Lasiorhinus*, by having a mandible with its greatest depth below M_4_, rather than M_3_ (Dawson, 1983). The Glenrock mandible cannot be compared with the Pleistocene *Vombatus hackettii* as corresponding mandibular elements have not been reported (Dawson, 1983). The mandible is heavily mineralised, suggesting considerable antiquity. *Vombatus ursinus* occurs in the region today.

#### Phalangeridae

*Trichosurus* sp. indet.

**Figure 6:**
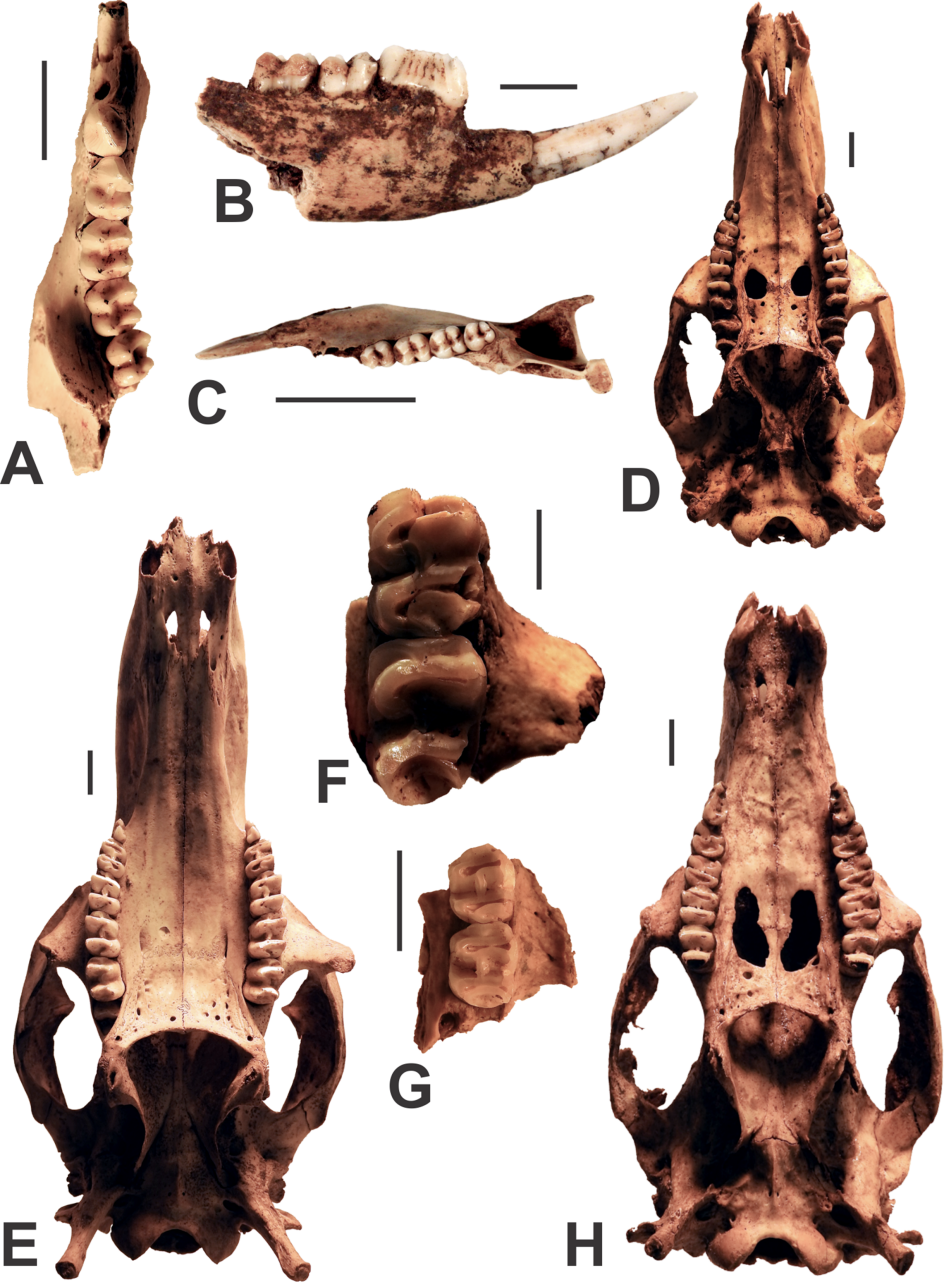
Diprotodontians from the Manning Karst Region. (A) *Trichosurus* sp. indet. left mandible (AODF0923). (B and C) *Bettongia gaimardi* right mandible in lateral view (AODF0924) and left mandible in occlusal view (AODF0925). (D) *Notamacropus rufogriseus* skull (AODF0926). (E) *Macropus giganteus giganteus* skull (AODF0927) with supernumerary molar. (F) *Macropus giganetus titan* maxilla (AODF0928). (G) *Petrogale* sp. maxilla (AODF0930). (H) *Wallabia bicolor* skull (AODF0931). Scale bars = 10 mm.

Brush-tailed possums are represented by isolated specimens from Fig Tree Cave and Death Trap caves including a left mandible (AODF0923; Fig. 6A). The short blade-like premolar is inflected outwards from the direction of the molar row. The molars are bilophodont. In terms of molar morphology, species of *Trichosurus* are somewhat similar to the rufus rat-kangaroo (*Aepyprymnus rufescens*), but isolated specimens from Glenrock lack a masseteric canal, a characteristic feature of kangaroos and their allies. AODF0923 is heavily mineralised, suggesting considerable antiquity, although brush-tailed possums continue to frequent the area today. This suggests that brush-tailed possums have occurred in the region for considerable time.

#### Macropodoidea

Bettongia gaimardi

Several mandibles are referred to the Eastern Bettong (e.g., AODF0924 and AODF0925; Figs. 6B and 6C, respectively). They are characterised by possessing a long, blade-like P_3_ that is angled anterobuccally relative to the molar row. The P_3_ has seven vertical ridgelets on the buccal side and eight on the lingual side. The molars are bunodont (but tending towards bilophodonty), with a masseteric canal in the jaw. The angular process at the posterior of the jaw is long and pointed, more so than in species of *Aepyprymnus*. The bones were found as surface specimens on shallow sandy deposits of two caves (Table 1) and are largely unmineralised suggesting that they are modern. Natural populations of the Eastern Bettong are extinct on the mainland, but it survives in Tasmania.

Notamacropus rufogriseus

The Red-necked Wallaby is represented by modern specimens within Red-naped Cave (e.g., AODF0926; Fig. 6D). They were identified on the basis of the medium-sized skull (relative to other macropodines) that has two large palatine-maxillary vacuities. The length of the P^3^ is less than that of the M^2^. Specimens were found only in caves, but on the surface of subterranean deposits. The bones are unmineralised, slightly discoloured, and are likely modern. Red-necked Wallabies are extant in the region.

Macropus giganteus giganteus

The modern Grey Kangaroo was recorded in several caves (Table 1), with additional fresh carcasses occasionally observed in various states of decay outside of the caves. They are characterised by a relatively large skull, small palatine vacuities, and variable fenestration on the palate (e.g., AODF0927; Fig. 6E). Subterranean specimens were occasionally observed, with some skeletons in near-complete articulation, suggesting minimal disturbance. The bones are generally white-ish in colour and unmineralised, indicating that such specimens are modern. Grey Kangaroos are extant in the region.

Macropus giganteus titan

The Giant Grey Kangaroo is represented by a partial maxilla that contains two teeth (AODF0928; Fig. 6F). Although the specimen lacks specific diagnostic features (i.e., premolars; Bartholomai, 1975), it is most similar in size to specimens previously attributed to *Macropus giganteus titan* rather than other extinct, but smaller-sized species within the genus. The specimen is mineralised and was recovered from the megafaunal deposit within Red-naped Cave. *Macropus giganteus titan* is the largest species recorded in the region, with individuals estimated to weigh up to 180 kg (Helgen et al., 2006).

Petrogale penicillata

Skeletal remains of Brush-tailed Rock Wallabies were recorded in several of the caves (Table 1). Identification of species of *Petrogale* is notoriously difficult considering the morphological similarities that exist between species (modern species are distinguished mostly on the basis of hair and soft tissue characters). However, given that the specimens are morphologically identical to *Petrogale penicllata*, and that it is the only known rock wallaby in the area, we consider the skeletal and dental remains referable to this species. Many of the bones are discoloured, but unmineralised suggesting that they are recent, although a fossil maxillary fragment (e.g., AODF0930; Fig. 6G) is recorded from the megafaunal deposit within Red-naped Cave. This record demonstrates that Brush-tailed Rock Wallabies have long frequented the Manning Karst Region.

Wallabia bicolor

A single Swamp Wallaby skull was observed on the surface of Red-naped Cave (AODF0931; Fig. 6H). The skull is medium-sized (relative to other macropodines) with two large palatine-maxillary vacuities. The P^3^ is the longer than any individual molar. Similar to the Red-necked Wallaby, the bones are discoloured but unmineralised, and likely modern. Extant populations are common in eastern Australia.

#### Microchiroptera

Microchiroptera gen. et sp. indet.

**Figure 7:**
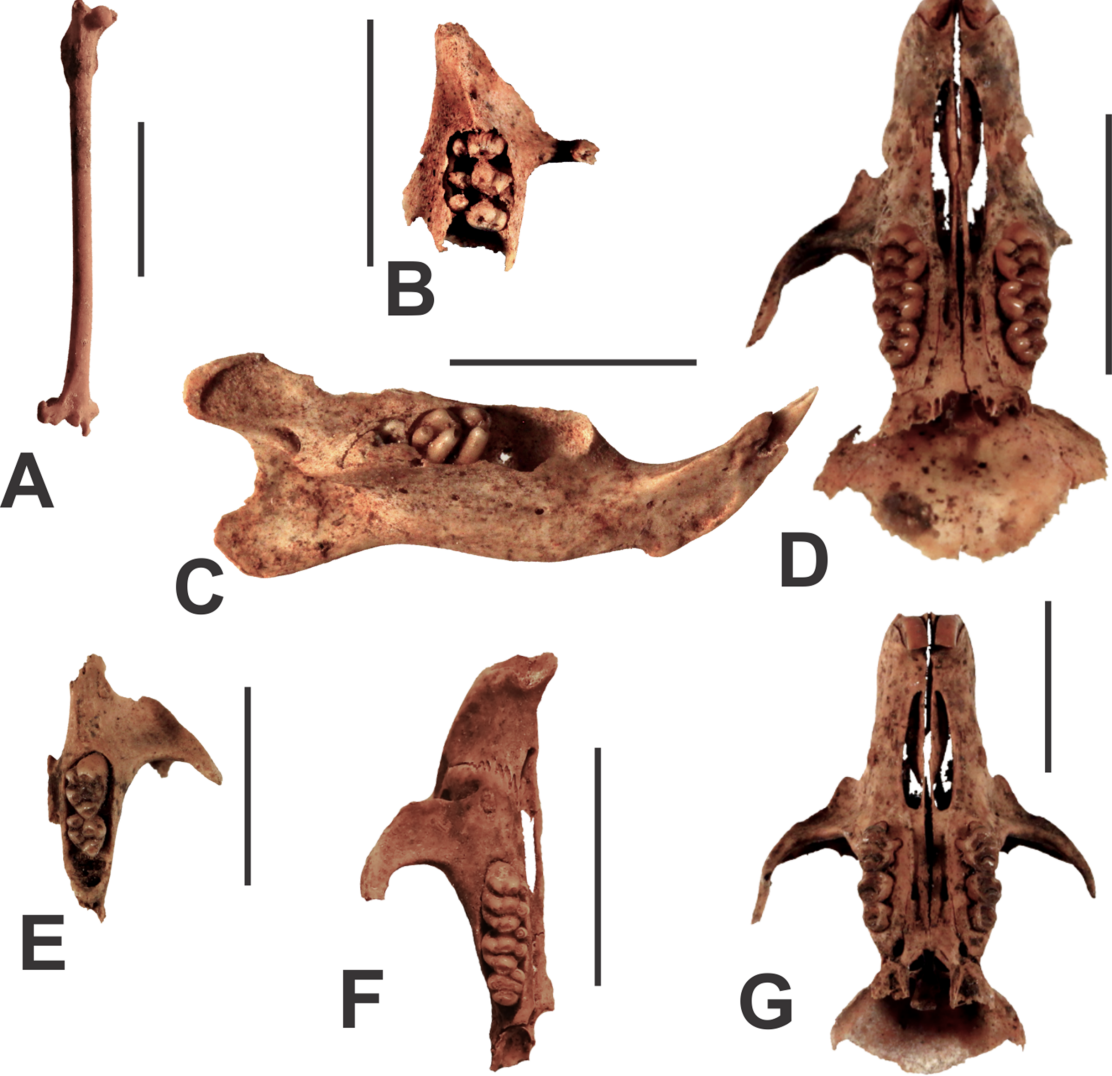
Endemic Australian placentals from the Manning Karst Region. Endemic Australian placentals from the Manning Karst Region. (A) Microchiropteran femur (AODF0932). (B–D) *Conilurus albipes* left maxilla (AODF0933) and left mandible (AODF0938). (D) *Pseudomys gracilicaudatus* skull (AODF0934). (E) *Pseudomys oralis* left maxilla (AODF0935). (F) *Rattus* sp. (*R. tunneyi*/*R. fuscipes*) right maxilla (AODF0936). (G) Muridae ge. et sp. indet. (AODF0937). Scale bars = 10 mm.

Post-cranial bones from bats were recorded as surface specimens in Westgate Cave (e.g., AODF0932; Fig. 7A). They lack features that allow diagnosis to the genus or species level. They are here referred to an indeterminate, but small-sized microchiropteran on the basis of their gracility in comparison to larger-bodied members of the suborder, such as megadermatids. They are also smaller than regionally extant fruit bats (megachiropterans). The bones are typically white in colour and unmineralisted, indicating their recent input into the cave deposits.

#### Muridae

Conilurus albipes

The White-footed Rabbit Rat is represented by a maxilla and mandible fragment (AODF0933 and AODF0938; Figs. 7B and 7C, respectively) found in Fig Tree Cave. This is the largest species of murid at Glenrock. It was identified on the basis of possessing high-crowned molars with distinct cusps (rather than transverse lophs common in other murids). Cusp T7 is well-developed on the M^1^ and M^2^. The bones are white in colour and unmineralised, suggesting that they are modern. The species is extinct having not been recorded alive since the 1860s.

Pseudomys gracilicaudatus

The Eastern Chestnut Mouse is represented by several maxillae (e.g., AODF0934; Fig. 7D) in Westgate Cave. In comparison to other species within the genus, *Pseudomys gracilicaudatus* is medium-sized and possesses relatively broad but low-crowned molars, anterior palatal foramena that extend past the anterior margin of the M^1^, and (commonly) an accessory cusp on M^1^. Specimens are unmineralised and collected from sandy surface deposits within the caves, suggesting that they are modern. Extant populations of the species have not been recorded in the Glenrock area.

Pseudomys oralis

The Hastings River Mouse is represented by maxillae (e.g., AODF0935; Fig. 7E) found on surface deposits in several caves in the region (Table 1). The species is relatively large in comparison to other members of the genus. It possesses high crowned cusps, with significant reduction of the T1 cusp on the M^1^. The anterior edge of the zygomatic plate is relatively straight. On the basis of preservation and occurrence within the caves, we suggest that the skeletal material of this species is modern. The Hastings River Mouse is found in forests in eastern New South Wales and south-east Queensland, but uncommonly encountered.

*Rattus* sp. indet. (*R. tunneyi* or *R. fuscipes*)

Species of *Rattus* are represented by partial skulls and mandibles from several of the region’s caves (Table 1). They are identified by their size (medium-sized in comparison to other Australian murids), possessing additional roots on most molars (including five on M^1^), sub-equal sized buccal and lingual cusps on M^1^, as well as a supplementary cusp on the antero-buccal corner of M_2_. Specimens from the Manning Karst Region (e.g., AODF0936; Fig. 7F) compare well with *R. tunneyi* (Pale Field Rat) and *R. fuscipes* (Bush Rat) in terms of molar dimensions and by possessing long anterior palatal foramena. These two species are difficult to separate purely on the basis of morphology. The specimens are unmineralised and were collected from the surface of deposits within the caves, suggesting that they are modern.

Muridae gen. et sp. indet.

One partial skull (AODF0937; Fig. 7G) could not be assigned to any known species of Australian rodent, either native or introduced. It is distinguished by a combination of the following features: medium-sized (similar in size to *Rattus* spp.) with a strongly constricted interorbital region, zygomatic plate that has a slightly concave anterior margin, broad anterior palatal foramena that are rounded posteriorly and end anterior of M^1^, deep furrows in the hard palate that connect the anterior palatal foramina to the posterior palatal foramina, and two lingual roots on M^1^. The constricted interorbital region is otherwise only seen in *Pseudomys auritus* (a very poorly known species), but the other features mentioned above are significantly different. The molars are worn, but the arrangement of cusps appear to resemble those of *Pseudomys australis*. It is possible that this specimen represents an undescribed species, but we reserve judgement on its taxonomy until more material comes to hand. The specimen was collected from the surface of Westgate Cave and is unmineralised, suggesting that it is modern.

#### Canidae

Vulpes vulpes

A skull of the Red Fox was observed as a surface specimen in Red-naped cave. The specimen is discoloured and unmineralised, indicating that it is modern. Foxes were introduced into Australia during the 19th century.

Canis familiaris

Numerous dog skeletons were observed on the surface of Ewe Beaut Cave. We attribute these specimens to wild dogs introduced by Europeans rather than the ‘purebred’ Dingo (both taxa are *Canis familiaris*, following Jackson et al., 2017) on the basis of them being slightly more brachiocephalic than the latter. The specimens are discoloured but unmineralised and clearly modern. A discarded margarine container was found partially buried adjacent to the dog skeletons. The use-by date printed on the bottom is July 1982, thus providing an approximate age of the skeletons.

#### Bovidae

Ovis aries

Sheep skeletons were observed in several of the caves, including Ewe Beaut Cave. Similar to other specimens from this cave floor (as others in the area), the skeletons are slightly discoloured and modern. Sheep were introduced to Australia in 1797.

#### Leporidae

Oryctolagus cuniculus

Skeletal material of Rabbits were observed as surface specimens on the floor of Lonesome Lair Cave. Rabbits are easily distinguished from Hares (*Lepus europaeus*) by possessing a more gently inclined vertical ramus relative to the horizontal ramus of the mandible. The bones are unmineralised and modern. Rabbits were introduced to the continent in the 18th century, but feral populations are thought to have become established in the mid-19th century.

### Taphonomy

Vertebrate remains were typically observed in one of three different sedimentary environments related to age and speleogenesis. The youngest remains consisted of surface finds, commonly within leaf-litter and other modern organics. These were typically unbroken or minimally damaged elements recovered in semi-articulated or disarticulated positions at the base of entry shafts. Fully articulated skeletons of medium-bodied mammals (i.e., dogs, sheep, kangaroos), or those almost fully articulated, were also commonly observed; however, these were almost always located away from the vertical entrances and indicate that falls into the vertical caves were not immediately fatal for many of these individuals.

Vertebrate remains belonging to small-bodied species were recovered in close association with larger mammals, as well as in smaller niches and crevices away from cave entrances. No obvious owl roost deposits were observed in any of the caves explored, and concentrations of small vertebrates never approached levels consistent with such accumulation modes. Indeed, as noted above, all cave entrances are at ground-level, thus, not conducive to roosting by owls. Additionally, we did not observe evidence of gnaw marks on the bones of small-bodied species suggestive of feeding from Ghost Bats (*Macroderma gigas*). It’s very likely that the small vertebrate accumulations represent the natural deaths of caverniculous or semi-caverniculous mammals and reptiles. The absence of small bird remains in the modern small fauna accumulations is noteworthy, and further supports this interpretation.

One particularly interesting accumulation included the skeletal remains of at least 15 individuals of wild dogs from the surface deposit of Ewe Beaut Cave. In contrast, large-boded herbivores (e.g., sheep and kangaroo) are far less common in the assemblage. Such skewed carnivore–herbivore ratios do not reflect natural communities, and are particularly rare occurrences in fossil assemblages, especially in Australia. We considered that the dogs may have been shot by local farmers with their carcasses thrown into the cave, but found no evidence of bullet holes on the skeletons. All of the wild dog individuals are adult. None of the bones of the dogs or other species possess gnaw marks. Those observations, combined with geomorphological considerations that show that the cave cannot be easily exited without assistance, suggest that the system does not function as a den. Thus, we suspect that the cave acted as a pitfall trap. It could be that a scenario such as envisaged for the La Brea Tar Pits carnivore-dominated assemblage (Shaw & Quinn, 1986; Stock & Harris, 1992) operates at Ewe Beaut Cave, whereby the smell of a decaying fallen animal (e.g., sheep, kangaroo) attracts an abundance of scavenging carnivores (in this case, wild dogs), some of which would also occasionally fall into the 11 m vertical pit. This would hypothetically lead to a greater incidence of carnivores in comparison to herbivores in the surface assemblage of the cave.

Vertebrate remains of much greater antiquity were observed in unconsolidated ferruginous clays and sandy silts, including species attributable to typical Pleistocene megafauna. In the case of Death Trap Cave, vertebrate remains were found in massive clay deposits in the lower chamber. Rill erosion (and in the main passage, transitioning to gully erosion) of the Death Trap Cave clay bed left large clasts and vertebrate remains on the floor of the cave. Some of these show evidence of moving downslope. In Red-naped Cave, similar sedimentological processes likely operated, with remnant deposits preserved in niches and joints in the wall, as well as the floor. Repeated drying and wetting of the clay likely fragmented many of the vertebrate fossil bone remains that were observed in Red-naped Cave. The megafauna deposit is stratigraphically intact, while the modern specimens from the cave (e.g. fox, sheep) have clearly never been buried and are found only on the surface of the deposit.

Cemented bone-bearing breccias were observed only in Fig Tree Cave, and consisted of deposits in two separate passages. At least one of these deposits is associated with flowstone formation, although both were preserved only as remnants on the cave wall and under a false floor, respectively. Both exhibit characteristics typical of bone breccias, namely preserving poorly sorted, fragmented vertebrate remains in fine-grained lithified ferruginous sandy-silts. Such deposits included both large and small mammal remains.

## Discussion

Our surveys of the Manning Karst Region have recovered over 30 vertebrate taxa from eight caves assessed for their palaeontological significance. The cave assemblages contain a variety of vertebrate taxa, but are skewed towards the preservation of mammals.

### Age of assemblages

Biochronological inferences based on the faunal assemblages suggest that the caves in the area have acted as pitfall traps since at least the mid-late Pleistocene. Megafaunal fossil material from Death Trap Cave and Red-naped Cave are likely the oldest in the area, having preserved extinct giant echidnas (Tachyglossidae gen. et sp. indet.), devils (*S. laniarius*), koalas (*Phascolarctos stirtoni*) and kangaroos (*Macropus giganteus titan*). It is clear that at least Red-naped Cave still acts as a pitfall trap considering that unmineralised skeletal remains of elapid snakes, Swamp Wallabies, Red Foxes, and sheep were recorded on the surface of the stratigraphically deeper megafaunal fossil deposit.

Other caves in the region, such as Fig Tree Cave, may also contain skeletal remains of significant antiquity considering the recovery of a fossil lower jaw of the extant ‘Tasmanian’ Devil (*S. harrisii*), a species thought to have suffered extinction on the mainland before 3,200 years ago (White et al., 2018). Placing a plausible maximum age on the deposit is not yet possible. In any case, similar to Red-naped Cave and Death Trap Cave, unmineralised surface specimens are prevalent in the system suggesting that the cave today continues to act as a site of accumulation of local vertebrates.

Other caves in the area, such as Lonesome Lair and Leaf Beetle Cave, represent comparative small cavernous systems that clearly do not function as significant pitfall traps for large-bodied vertebrates. Taxa recorded in such caves include unmineralised remains of various macropodids (e.g. Rock Wallabies) and introduced exotics (e.g. European Rabbits). Such assemblages are historic (<200 years).

### Megafauna

Several megafauna were recorded in the caves at Glenrock, including the giant extinct Stirton’s Koala, *Phascolarctos stirtoni*. In general, koalas are rare components of the Australian fossil record (Black, Louys & Price, 2014), and this is especially true of *Phascolarctos stirtoni*. The species was described on the basis of a single maxillary fragment (Bartholomai, 1968), with only scant specimens referred to it since (Price, 2008). Although the species is known only from maxillary and mandibular remains, the new record of AODF0920 represents an important addition to the hypodigm to the species, and is also the first record of the taxon in New South Wales. The discovery demonstrates that the species was widespread across eastern Australia during the Pleistocene, from central eastern Queensland to southeastern South Australia (Price et al., 2009a).

Similarly, skeletal remains of echidnas are particularly rare in Australia (Griffiths, Wells & Barrie, 1991). Pleistocene records of large-bodied echidnas have previously been reported from the Darling Downs of southeast Queensland (Price & Webb, 2006), Wellington Caves of central New South Wales (Murray, 1978), Naracoorte of southeastern South Australia (Reed & Bourne, 2009), Tasmania (Murray, 1978), and southwestern Western Australia (Griffiths, Wells & Barrie, 1991). The new record from the Manning Karst Region is an important addition to the known geographic range of these giant echidnas, extending their distribution over 250 km further east of the nearest echidna-bearing Pleistocene deposit (Wellington Caves).

### Baseline fauna and conservation insights

The presence of fossilised remains of devils (species of *Sarcophilus*) in the region has important implications for their conservation. Today, the extant Tasmanian Devil is plagued by Devil Facial Tumour Disease (McCallum et al., 2007). In combination with increased anthropogenic pressures through habitat disturbance and vehicular strikes, natural populations have declined dramatically over the past couple of decades. Plans for reintroducing disease-free devils back to the mainland have been considered (Louys et al., 2014), not only as a way of prolonging the survivorship of the species, but as a potential biological control of medium-sized introduced (and feral) carnivores such as foxes and cats (Hollings et al., 2014). In 2011, a breeding colony of disease-free devils was established at Barrington Tops, approximately 15 km south of the Glenrock area (Payne, 2012). The recent successes of the breeding program, combined with palaeontological information indicating the local occurrence of devils in the geologically recent past, suggests that region has the geography and climatic characteristic conducive to supporting populations of re-introduced Tasmanian Devils. The key question, however, is whether devils could survive the combination of competition from wild dogs (including Dingoes), as well as modern local anthropogenic impacts.

Numerous extant small-bodied species are recorded in the caves of the Manning Karst Region that represent significant new spatial and temporal extensions. For instance, the Southern Brown Bandicoot (*I. obesulus*), thought to be restricted to areas 250 km further south (Paull, 2008), was recorded as unmineralised surface specimens in several of the caves. Although fossil populations are found at the Wellington and Jenolan Caves (Dawson & Augee, 1997; Morris et al., 1997) indicating significant longevity of the species in New South Wales, the specimens from Glenrock are clearly modern and suggest that the species survived locally until relatively recently. It is possible that the Southern Brown Bandicoot still occurs locally considering the nature of preservation and occurrence as surface specimens in several of the region’s caves. However, this can only be determined (or excluded) following intensive trapping and surveying of small-bodied mammals living in the area today, something, that is, yet to happen. If such hypothetical studies failed to capture Southern Brown Bandicoots, the cavernous records would at least suggest that the species survived into historic times (i.e. post-European colonisation), but was never recorded alive prior to its local extirpation. If so, then ecological surveys used to determine the geographic range contraction of modern small-bodied mammals since European colonisation substantially underestimate the true historic geographic range of their occurrence, and the complete range of habitat tolerances of a given species (Fusco, McDowell & Prideaux, 2016).

Similarly, other small-bodied mammals have also been recorded as unmineralised surface specimens in the Glenrock caves, but are now locally extinct, including the Eastern Bettong (*Bettongia gaimardi*), Eastern Chestnut Mouse (*Pseudomys gracilicaudatus*), and White-footed Rabbit Rat (*Conilurus albipes*). In the historic period, the Eastern Bettong was widespread with populations across southeastern Australia and Tasmania, however, it is now extirpated on the mainland. Similar to the Tasmanian Devil, captive breeding programs have been established on the mainland, with individuals already released into fenced enclosures at Tidbinbilla Nature Reserve and Mulligan’s Flats Woodland Sanctuary in the Australian Capital Territory (Portas et al., 2014).

Modern surveys of the Eastern Chestnut Mouse indicate that the species occurs in two broad but disjunct areas, including much of eastern Queensland and northeast New South Wales, and around the Jervis Bay area of central eastern New South Wales (Meek & Triggs, 1998). The fossil record clearly indicates a significantly wider range in the recent past (Morris et al., 1997).

Perhaps most significantly, the White-footed Rabbit Rat is now totally extinct despite having been widespread in southeastern Australia at the time of European colonisation. Moreover, it had a long history in the region having been recorded in various Pleistocene deposits of northeast and central eastern Queensland (Cramb & Hocknull, 2010), The Joint and Russenden Caves of southeast Queensland (Price et al., 2009b), and Wellington (Dawson & Augee, 1997) and Jenolan Caves (Morris et al., 1997) of New South Wales, including as surface specimens at each location (as well as Glenrock). Thus, its evolutionary history suggests it had a wide range of climatic and habitat tolerances. Despite having been so widespread and apparently very common in the early days of European colonisation, they’ve since proven particularly vulnerable to subsequent anthropogenic impacts, although the precise driver of their loss remains unknown (Dixon, 2008).

The finding of a partial skull referable only to a species of rodent (Muridae gen. et sp. indet.; AODF0937 from Westgate Cave; Fig. 7G) remains somewhat puzzling. Despite being relatively complete, we have not been able place it within any other extant genus of Australian rodent. The significance of this yet to be determined, but it remains possible that the specimen represents a new species and genus. If that is the case and judging by the preservation (unmineralised and collected from the surface of a cave floor), this specimen may represent an undescribed rodent species and victim of the most recent wave of small-bodied mammal extinctions in Australia in historic times. That is, it may be a taxon that existed until relatively recently, but suffered extinction prior to its documentation as a living organism. Additional specimens would be required to test that hypothesis.

At a community level, our results indicate that a diverse range of small-bodied digging species have suffered extinction in the Manning Karst Region, at least since the late Pleistocene, but especially during the historic period. Those taxa include several of the abovementioned species such as bettongs, bandicoots, and rodents, as well as large-bodied echidnas. Such mammals are commonly considered to be ‘forest engineers’, having clear and important roles dispersers of mycorrhizal fungi and seeds, and bioturbators of soils such as to alter its chemical and physical properties (Fleming et al., 2014). Each of those factors are critical for sustaining, or even promoting increases in, local vegetation and associated biodiversity. Removal of such taxa from an environment may lead to loss of ecosystem function, with obvious impacts on long-term biodiversity (Martin, 2003). Indeed, their extirpation has already likely led to a decrease in the functionality of contemporary local ecosystems.

That observation leads back to an important earlier point regarding the potential of reintroductions of species to the mainland such as Tasmanian Devils and Eastern Bettongs. Such translocation strategies will only be feasible if they go beyond simple considerations of current and suitable climate and habitat types, but take a total ecosystem approach. Critically, this must incorporate insights from historical biology and the functionality of ecosystems prior to, and in the early days of, European colonisation. We have demonstrated that such insights can be gleaned from the fossil record and that caves have a major role in that regard.

## Conclusions

There is great potential for future palaeontological work within the caves in the Manning Karst Region. New caves are being discovered each year, and in general, speleologists are not specifically searching for, nor trained in the identification of, fossils and bones. Significantly, many of the recently discovered caves were revealed as a result of digging; they clearly have not been open to the surface as natural animal traps for millennia. Hence, any bones found in the newly excavated caves have the potential to be very old. This interference is supported by observations from Death Trap Cave (entrance excavated in 2010) that contains fossilised remains of native species to the exclusion of recently introduced exotics.

The cave assemblages in the Manning Karst Region provide clear evidence for species extinction and faunal turnover, starting with the loss of megafaunal taxa sometime since the late Pleistocene. Faunal loss continued through to today, with numerous local extirpations and even extinctions of small-bodied mammals since European colonisation, along with the replacement by introduced exotics. The shifting faunal baseline, especially in recent times, has likely led to severe disruption of local ecosystem function. Understanding the faunal baseline is fundamental for assessing the degree of loss or change in modern ecosystems. Such insights are crucial in setting goals and benchmarks for conservation.

Many caves remain to be explored for their palaeontological significance, not only in the Manning Karst Region, but across vast expanses of eastern Australia. Such caves likely contain important fossil assemblages noteworthy of greater scrutiny, but the work can only go ahead following the support of relevant research funding agencies, not to mention the collaboration and cooperation of speleologists, palaeontologists, and land managers (and owners) alike.

## Supplemental Information

10.7717/peerj.6099/supp-1 Supplemental Information 1 Map of GR1 Fig tree Cave (modified from Pinnock, 1987). A. Elevation view. B. Plan view. Magnetic north indicated by arrow. Scale bars = 5 m. Bone symbol = approximate area where skeletal material found. Red dashed-line = section line from which angle of elevation is drawn. Original map surveyed and drawn by Adrian Ridgley, Richard Pinnock, and Michael Ryan.

10.7717/peerj.6099/supp-2 Supplemental Information 2 Map of GR2 Clunk, Clunk, Bang, Pot Cave (modified from Pinnock, 1987). A. Elevation view. B. Plan view. Magnetic north indicated by arrow. Scale bars = 5 m. Bone symbol = approximate area where skeletal material found. Red dashed-line = section line from which angle of elevation is drawn. Original map surveyed and drawn by Richard Pinnock, Adrian Ridgley, and Lionel Hine.

10.7717/peerj.6099/supp-3 Supplemental Information 3 Map of GR5 Ewe Beaut Cave (modified from Pinnock, 1987). A. Elevation view. B. Plan view. Magnetic north indicated by arrow. Scale bars = 5 m. Bone symbol = approximate area where skeletal material found. Red dashed-line = section line from which angle of elevation is drawn. Original map surveyed and drawn by Richard Pinnock, Rob Crncovic, and Chris Hine.

10.7717/peerj.6099/supp-4 Supplemental Information 4 Map of GR17 Red-naped Cave (modified from Pinnock, 1987). A. Elevation view. B. Plan view. Magnetic north indicated by arrow. Scale bars = 5 m. Bone symbol = approximate area where skeletal material found. Red dashed-line = section line from which angle of elevation is drawn. Original map surveyed and drawn by Adrian Ridgley, Shane Wilcox, and Richard Pinnock.

10.7717/peerj.6099/supp-5 Supplemental Information 5 Map of GR18 Westgate Cave (modified from Pinnock, 1987). A. Elevation view. B. Plan view. Magnetic north indicated by arrow. Scale bars = 5 m. Bone symbol = approximate area where skeletal material found. Red dashed-line = section line from which angle of elevation is drawn. Original map surveyed and drawn by Ian Hine, Adrian Ridgley, Lionel Hine, Chris Hine, and Richard Pinnock.

10.7717/peerj.6099/supp-6 Supplemental Information 6 Map of GR36 Lonesome Lair Cave (modified from Pinnock, 1987). A. Elevation view. B. Plan view. Magnetic north indicated by arrow. Scale bars = 5 m. Bone symbol = approximate area where skeletal material found. Red dashed-line = section line from which angle of elevation is drawn. Original map surveyed and drawn by Graeme Kates and Chris Hine.

10.7717/peerj.6099/supp-7 Supplemental Information 7 Map of GR92 Leaf Beetle Cave (modified from Pinnock, 1987), plan view. Magnetic north indicated by arrow. Scale bar = 5 m. Bone symbol = approximate area where skeletal material found. Original map surveyed and drawn by Adrian Ridgley and Richard Pinnock.

10.7717/peerj.6099/supp-8 Supplemental Information 8 Map of GR124 Death Trap Cave (modified from Smith, 2011b). A. Elevation view. B. Plan view (upper level). C. Plan view (lower level). Magnetic north indicated by arrow. Scale bars = 5 m. Bone symbol = approximate area where skeletal material found. Red dashed-line = section line from which angle of elevation is drawn. Original map surveyed and drawn by Garry Smith, Geoffrey McDonnell, and Jodie Rutledge.

## Additional Institutional Abbreviations

AODF: Australian Age of Dinosaurs Fossil
AODL: Australian Age of Dinosaurs Locality
SAMP: South Australian Museum Palaeontology Collection

## Additional Information and Declarations

### Data Availability

The following information was supplied regarding data availability: All new specimens figured and mentioned in this study are registered in the collections of the Australian Age of Dinosaurs Museum of Natural History, Winton, Queensland, Australia. All fossils will be made available to bonafide researchers upon publication of this study. Contact Trish Sloan (Operations Manager) at operations@aaod.com.au to request this information. The following specimens have been accessioned into the fossil collections of the museum (specific details about each specimen are given in the main text): AODF0907–AODF0938.
