# Supplementary figures and images for: Shifting faunal baselines through the Quaternary revealed by cave fossils of eastern Australia
Source: PeerJ. 2019 Jan 22;6:e6099. doi: 10.7717/peerj.6099 (PMC6346992; doi:10.7717/peerj.6099)

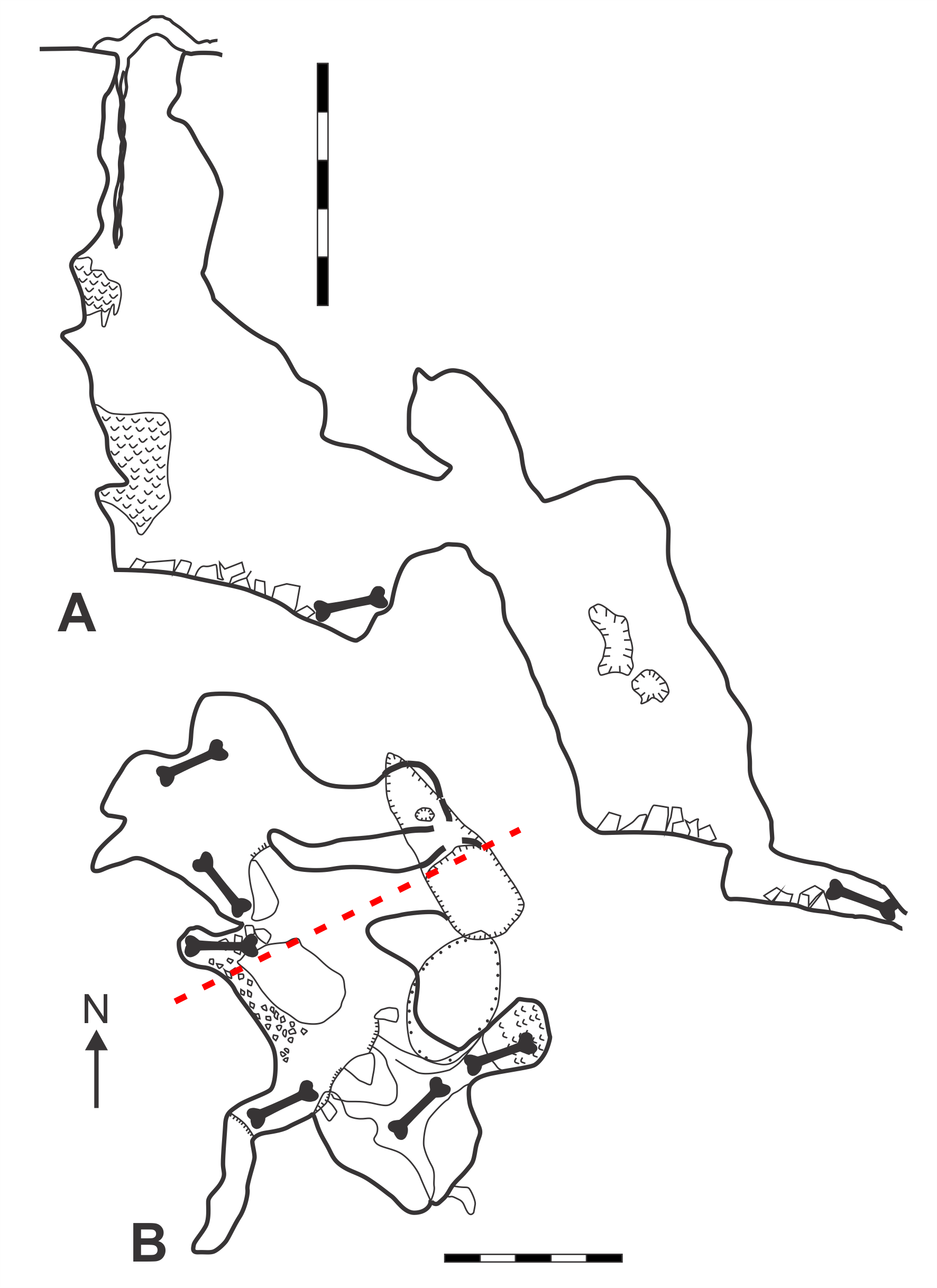

Supplement: Supplemental Information 1 — A. Elevation view. B. Plan view. Magnetic north indicated by arrow. Scale bars = 5 m. Bone symbol = approximate area where skeletal material found. Red dashed-line = section line from which angle of elevation is drawn. Original map surveyed and drawn by Adrian Ridgley, Richard Pinnock, and Michael Ryan. [file peerj-07-6099-s001.png]

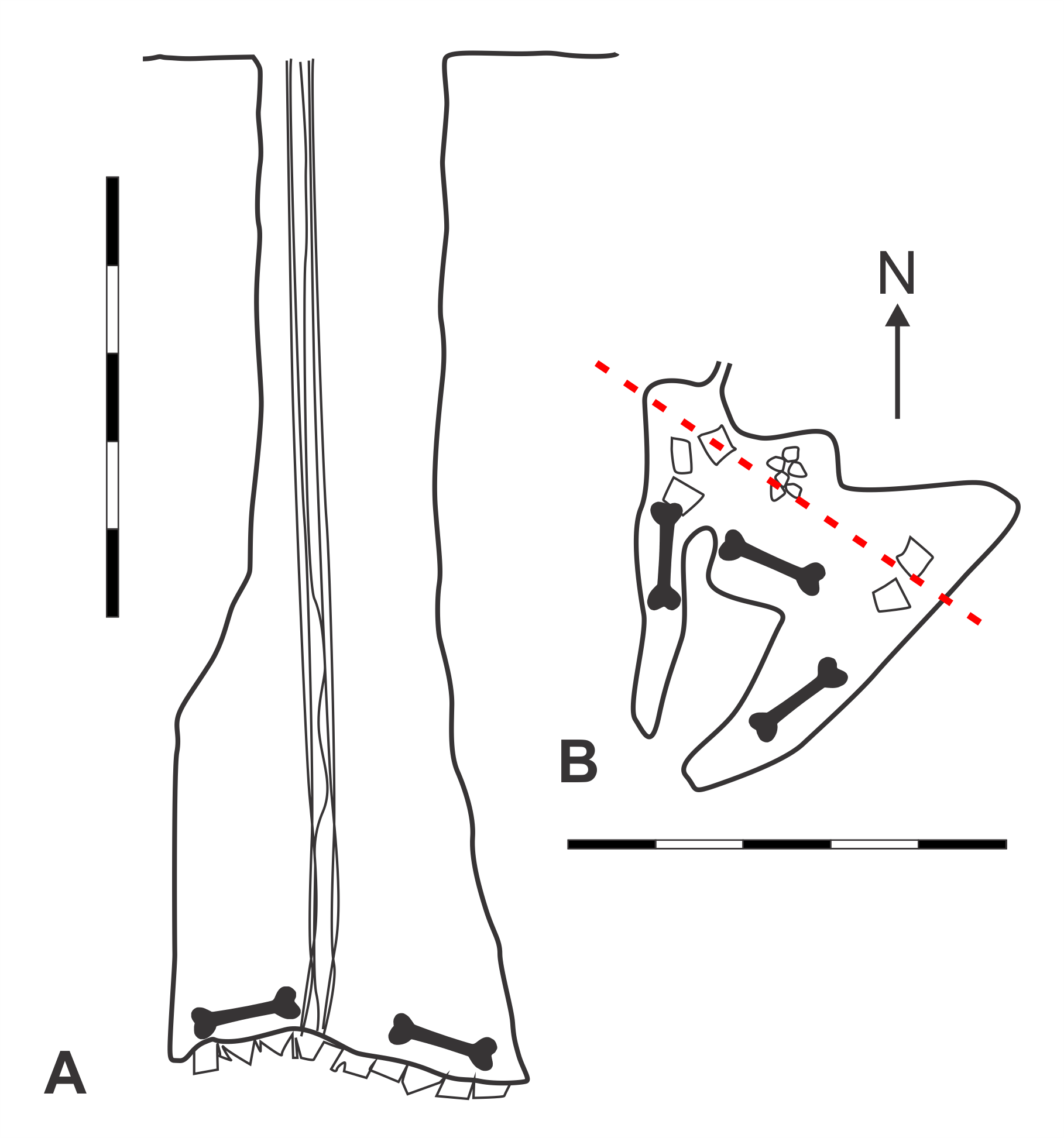

Supplement: Supplemental Information 2 — A. Elevation view. B. Plan view. Magnetic north indicated by arrow. Scale bars = 5 m. Bone symbol = approximate area where skeletal material found. Red dashed-line = section line from which angle of elevation is drawn. Original map surveyed and drawn by Richard Pinnock, Adrian Ridgley, and Lionel Hine. [file peerj-07-6099-s002.png]

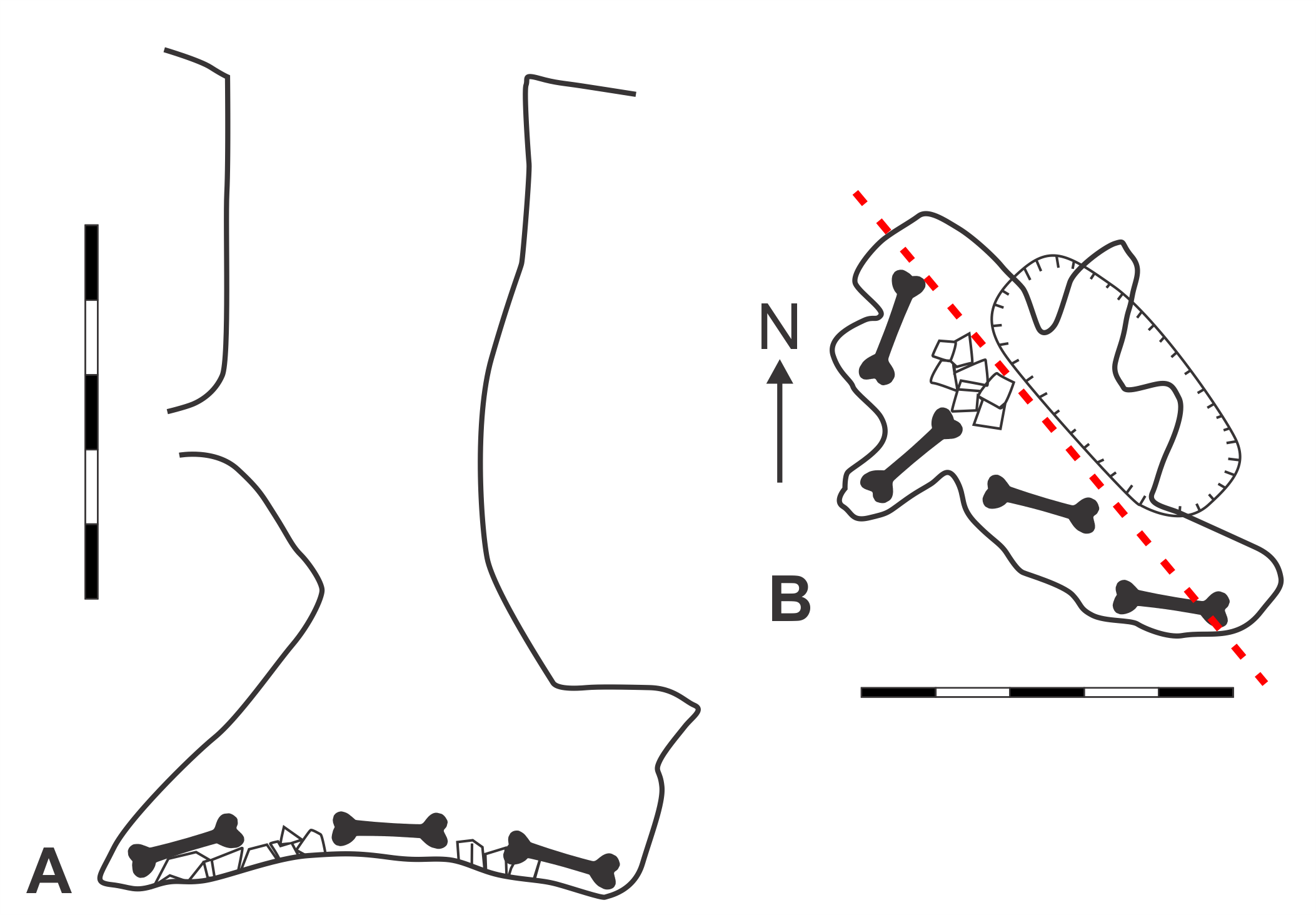

Supplement: Supplemental Information 3 — A. Elevation view. B. Plan view. Magnetic north indicated by arrow. Scale bars = 5 m. Bone symbol = approximate area where skeletal material found. Red dashed-line = section line from which angle of elevation is drawn. Original map surveyed and drawn by Richard Pinnock, Rob Crncovic, and Chris Hine. [file peerj-07-6099-s003.png]

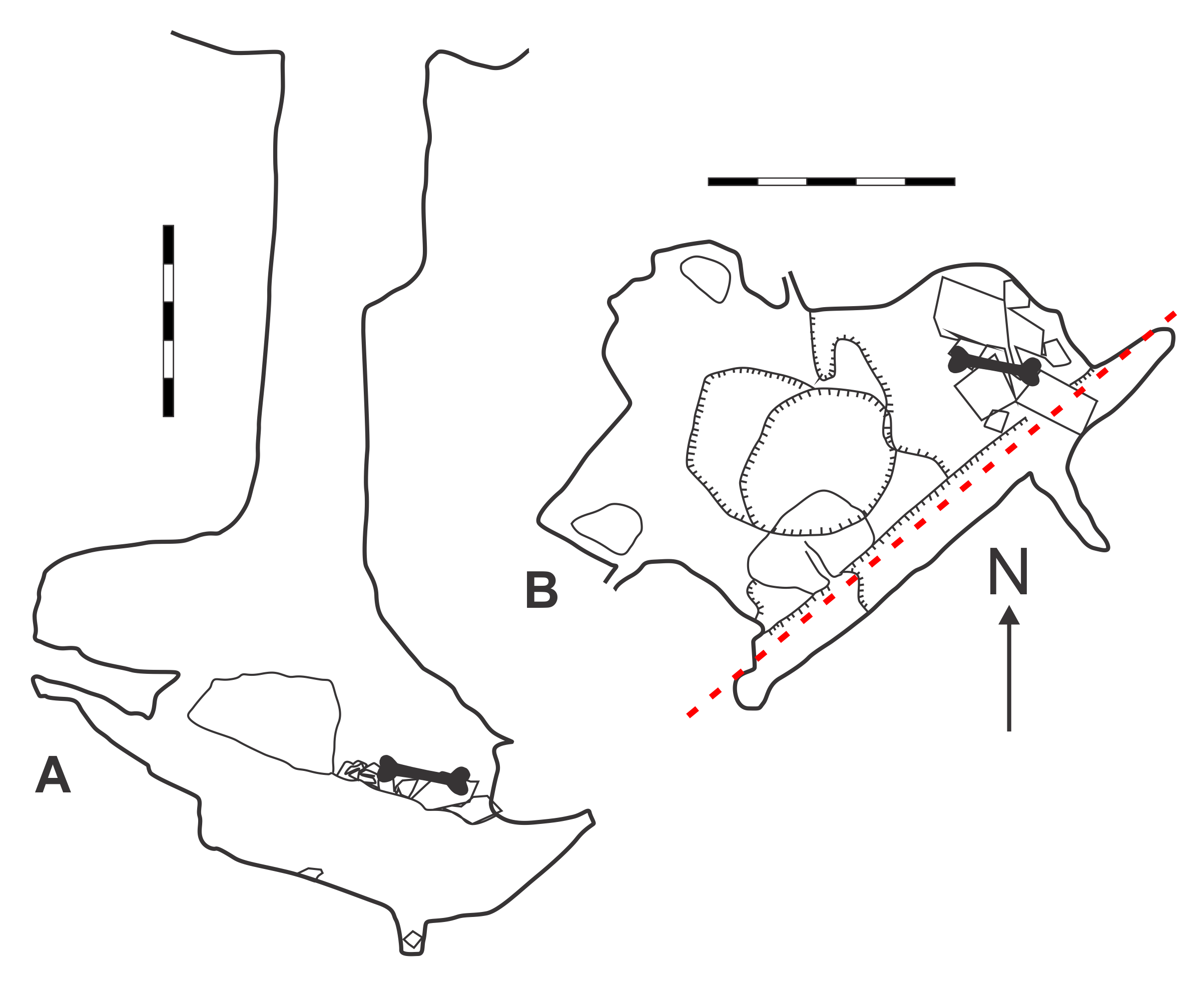

Supplement: Supplemental Information 4 — A. Elevation view. B. Plan view. Magnetic north indicated by arrow. Scale bars = 5 m. Bone symbol = approximate area where skeletal material found. Red dashed-line = section line from which angle of elevation is drawn. Original map surveyed and drawn by Adrian Ridgley, Shane Wilcox, and Richard Pinnock. [file peerj-07-6099-s004.png]

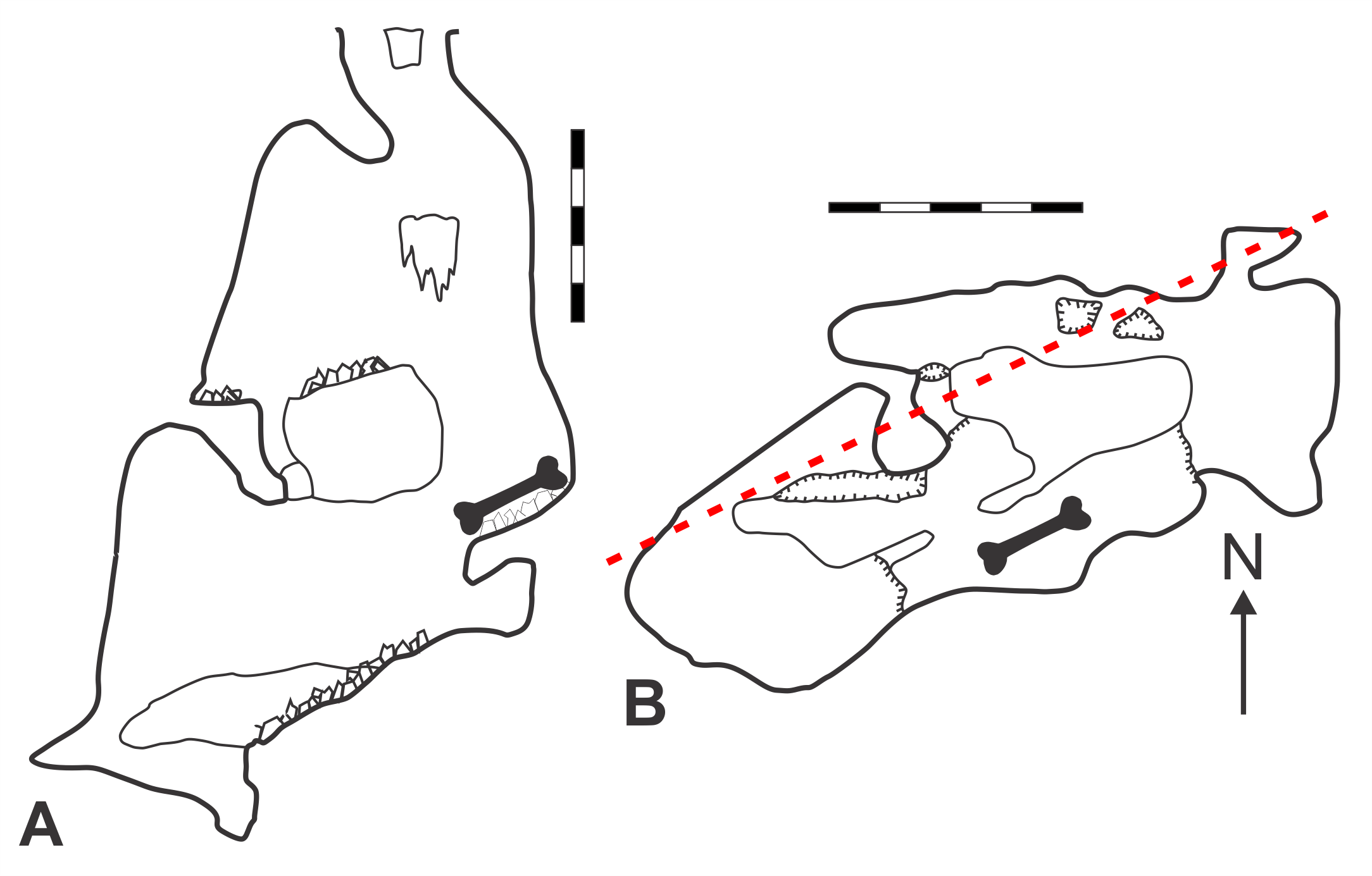

Supplement: Supplemental Information 5 — A. Elevation view. B. Plan view. Magnetic north indicated by arrow. Scale bars = 5 m. Bone symbol = approximate area where skeletal material found. Red dashed-line = section line from which angle of elevation is drawn. Original map surveyed and drawn by Ian Hine, Adrian Ridgley, Lionel Hine, Chris Hine, and Richard Pinnock. [file peerj-07-6099-s005.png]

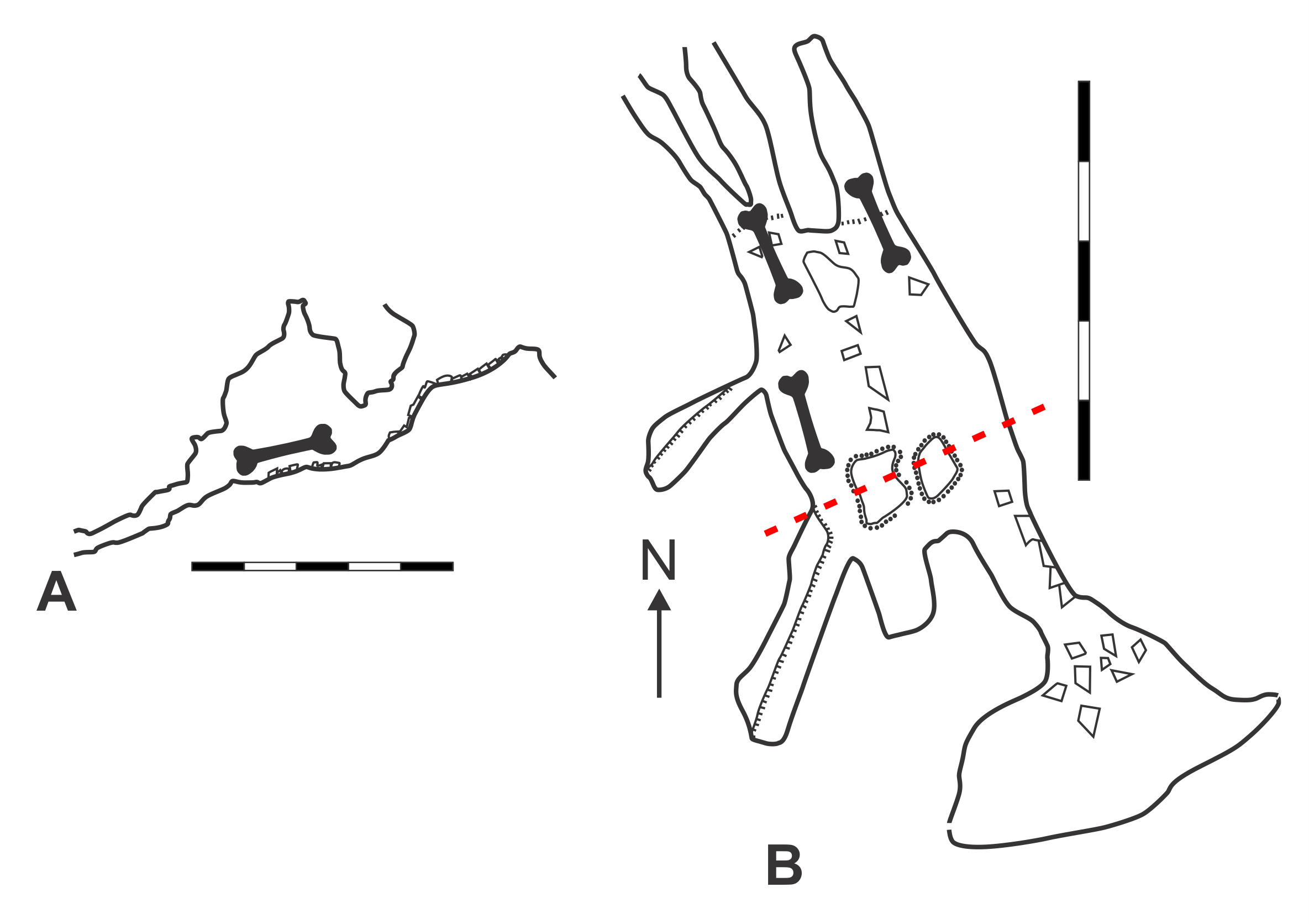

Supplement: Supplemental Information 6 — A. Elevation view. B. Plan view. Magnetic north indicated by arrow. Scale bars = 5 m. Bone symbol = approximate area where skeletal material found. Red dashed-line = section line from which angle of elevation is drawn. Original map surveyed and drawn by Graeme Kates and Chris Hine. [file peerj-07-6099-s006.png]

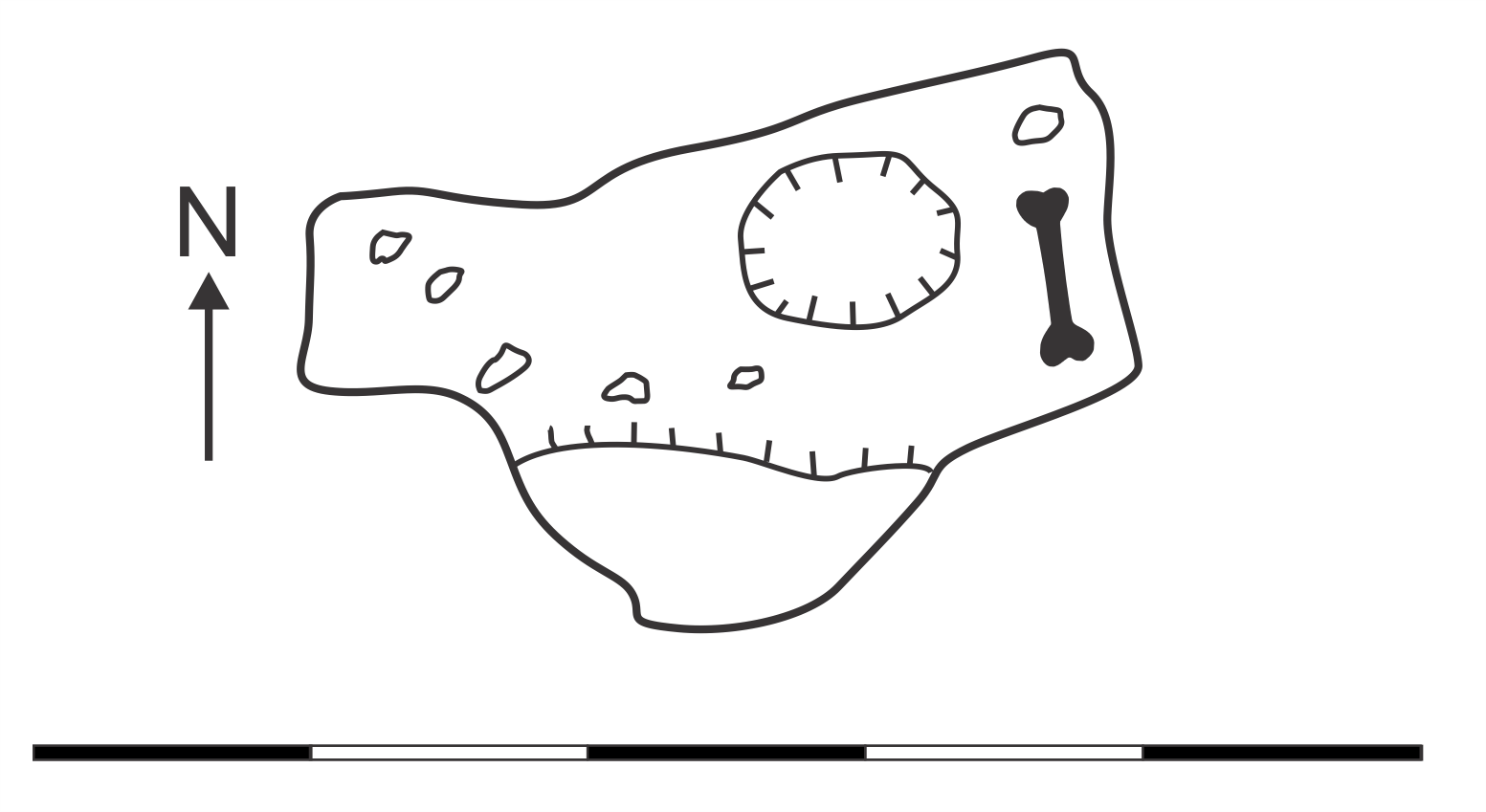

Supplement: Supplemental Information 7 — Magnetic north indicated by arrow. Scale bar = 5 m. Bone symbol = approximate area where skeletal material found. Original map surveyed and drawn by Adrian Ridgley and Richard Pinnock. [file peerj-07-6099-s007.png]

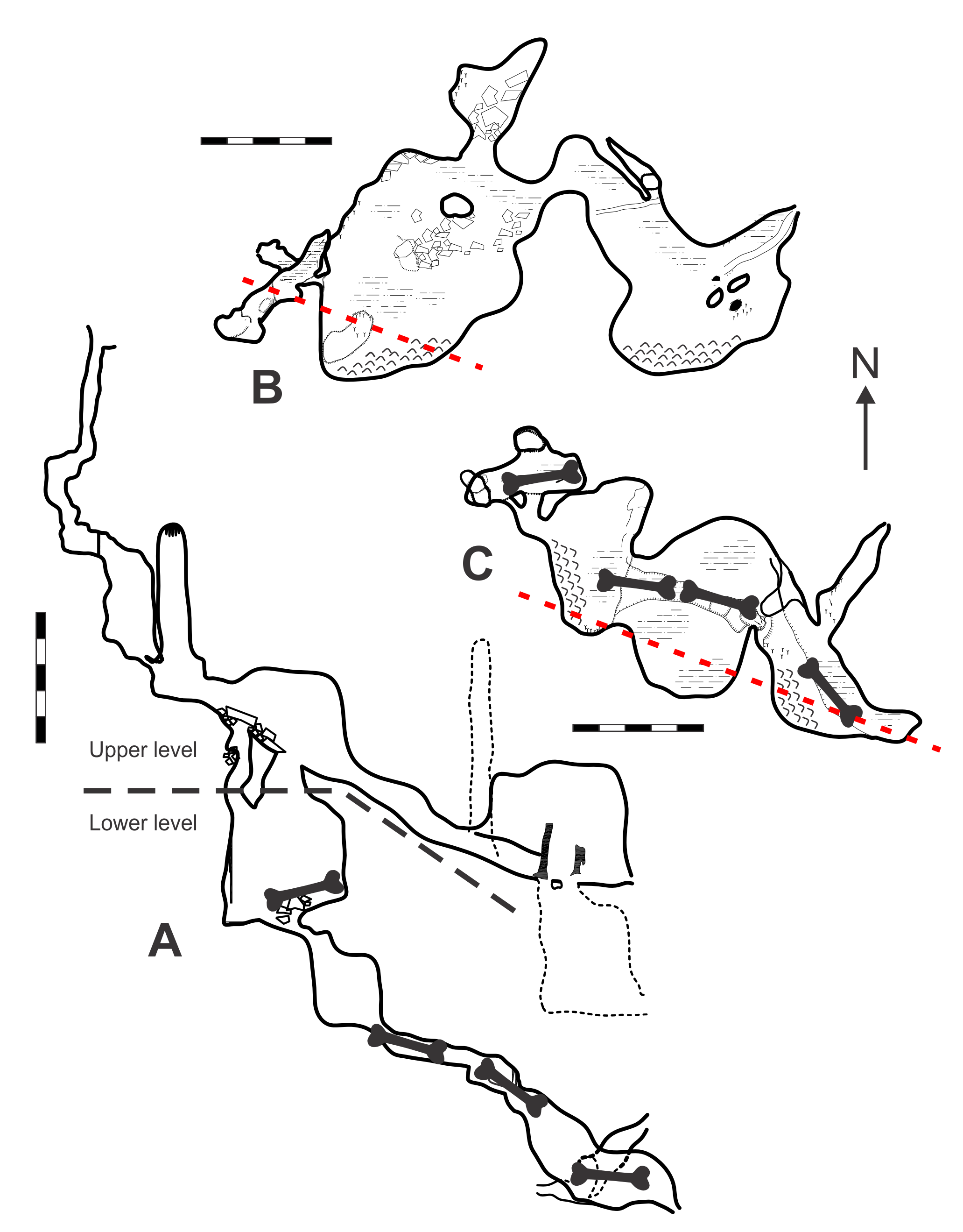

Supplement: Supplemental Information 8 — A. Elevation view. B. Plan view (upper level). C. Plan view (lower level). Magnetic north indicated by arrow. Scale bars = 5 m. Bone symbol = approximate area where skeletal material found. Red dashed-line = section line from which angle of elevation is drawn. Original map surveyed and drawn by Garry Smith, Geoffrey McDonnell, and Jodie Rutledge. [file peerj-07-6099-s008.png]
